# Defects of mitochondria-lysosomes communication induce secretion of mitochondria-derived vesicles and drive chemoresistance in ovarian cancer cells

**DOI:** 10.1186/s12964-024-01507-y

**Published:** 2024-03-06

**Authors:** Sinforosa Gagliardi, Marco Mitruccio, Riccardo Di Corato, Roberta Romano, Alessandra Aloisi, Rosaria Rinaldi, Pietro Alifano, Flora Guerra, Cecilia Bucci

**Affiliations:** 1 Department of Biological and Environmental Sciences and Technologies, University of Salento, Via Provinciale Lecce-Monteroni n. 165, Lecce, 73100 Italy; 2 Department of Experimental Medicine, University of Salento, Via Provinciale Lecce-Monteroni n. 165, Lecce, 73100 Italy; 3 Institute for Microelectronics and Microsystems (IMM), CNR, Via Monteroni, Lecce, 73100 Italy; 4 Center for Biomolecular Nanotechnologies, Istituto Italiano di Tecnologia, Arnesano, 73010 Italy; 5 Department of Mathematics and Physics “E. De Giorgi”, University of Salento, Via Monteroni, Lecce, 73100 Italy; 6 Scuola Superiore ISUFI, University of Salento, Via Monteroni, University Campus, Lecce, 73100 Italy

**Keywords:** RAB7, Mitochondria-derived vesicles, Extracellular vesicles, Cisplatin chemoresistance, Ovarian cancer

## Abstract

**Background:**

Among the mechanisms of mitochondrial quality control (MQC), generation of mitochondria-derived vesicles (MDVs) is a process to avoid complete failure of mitochondria determining lysosomal degradation of mitochondrial damaged proteins. In this context, RAB7, a late endocytic small GTPase, controls delivery of MDVs to late endosomes for subsequent lysosomal degradation. We previously demonstrated that RAB7 has a pivotal role in response to cisplatin (CDDP) regulating resistance to the drug by extracellular vesicle (EVs) secretion.

**Methods:**

Western blot and immunofluorescence analysis were used to analyze structure and function of endosomes and lysosomes in CDDP chemosensitive and chemoresistant ovarian cancer cell lines. EVs were purified from chemosensitive and chemoresistant cells by ultracentrifugation or immunoisolation to analyze their mitochondrial DNA and protein content. Treatment with cyanide m-chlorophenylhydrazone (CCCP) and RAB7 modulation were used, respectively, to understand the role of mitochondrial and late endosomal/lysosomal alterations on MDV secretion. Using conditioned media from chemoresistant cells the effect of MDVs on the viability after CDDP treatment was determined. Seahorse assays and immunofluorescence analysis were used to study the biochemical role of MDVs and the uptake and intracellular localization of MDVs, respectively.

**Results:**

We observed that CDDP-chemoresistant cells are characterized by increased MDV secretion, impairment of late endocytic traffic, RAB7 downregulation, an increase of RAB7 in EVs, compared to chemosensitive cells, and downregulation of the TFEB-mTOR pathway overseeing lysosomal and mitochondrial biogenesis and turnover. We established that MDVs can be secreted rather than delivered to lysosomes and are able to deliver CDDP outside the cells. We showed increased secretion of MDVs by chemoresistant cells ultimately caused by the extrusion of RAB7 in EVs, resulting in a dramatic drop in its intracellular content, as a novel mechanism to regulate RAB7 levels. We demonstrated that MDVs purified from chemoresistant cells induce chemoresistance in RAB7-modulated process, and, after uptake from recipient cells, MDVs localize to mitochondria and slow down mitochondrial activity.

**Conclusions:**

Dysfunctional MQC in chemoresistant cells determines a block in lysosomal degradation of MDVs and their consequent secretion, suggesting that MQC is not able to eliminate damaged mitochondria whose components are secreted becoming effectors and potential markers of chemoresistance.

**Supplementary Information:**

The online version contains supplementary material available at 10.1186/s12964-024-01507-y.

## Background

In recent years, mitochondria-derived vesicles (MDVs) have attracted the attention of researchers as numerous studies have identified mitochondrial signatures in extracellular vesicles (EVs) as markers of neurodegenerative diseases such as Parkinson’s and Alzheimer’s disease, and of aging [1–3]. In fact, these conditions correlate with mitochondrial dysfunction and alteration of mitochondrial quality check (MQC) mechanisms. In addition, MDVs are considered as part of the MQC pathway [4, 5]. Indeed, in the first instance, MDVs are generated to eject damaged mitochondrial proteins in order to avoid the complete failure of the organelles. Thus, MDVs are considered the first round of mitochondria defense and their generation occurs as soon as reactive oxygen species (ROS) are produced. Upon complete depolarization and organelle dysfunction, the mechanism of MDV biogenesis switches toward mitophagy [6]. Once generated for the elimination of oxidized cargoes, MDVs undergo fusion with late endocytic compartments to final lysosomal degradation.

In recent years, emerging evidence highlighted the importance of organelle intercommunication to guarantee cellular homeostasis [7]. In particular, several works described the consequences of dysfunctional mitochondria-lysosome crosstalk in neurodegeneration [8, 9]. The establishment of mitochondria-lysosome contact sites has been included among the mechanisms participating in MQC as an additional layer of quality check, involving crosstalk between the two organelles and culminating in the release of EVs of mitochondrial origin, the MDVs. Today, the mechanism of MDV secretion is still poorly understood and, in particular, the role of MDVs in cancer and chemoresistance has not yet been investigated.

Ovarian cancer is the most lethal gynecologic cancer [10]. The current standard therapy for patients is based on cisplatin (CDDP), but resistance to therapy is one of the main issues associated with chemotherapy treatment. CDDP crosslinks DNA and induces DNA-damage that elicits DNA repair, which in turn activates mitochondria-mediated apoptosis when DNA repair capability is saturated [11, 12]. Many mechanisms of resistance to CDDP have been proposed, including reduced uptake or increased efflux of the drug, detoxification, increased DNA repair and inhibition of apoptosis [13–16]. Epigenetic mechanisms are also involved [15, 17, 18]. In previous work, we demonstrated that impairment of the late endocytic pathway is a common feature of the chemoresistant phenotype of several gynecologic cancer cell lines. In particular, we found that the small GTPase RAB7A (Ras-related in brain-7 A, from now on referred to as RAB7), the main regulator of the late endocytic pathway [19–22], is downregulated in all chemoresistant cells analyzed, and that RAB7 depletion increases CDDP-resistance while RAB7 overexpression decreases it. Moreover, downregulation of RAB7 results in the switch from CDDP-sensitivity to CDDP-resistance by increasing the secretion of CD9 and CD81 positive EVs and the efflux of CDDP through EVs [23].

Importantly, literature data indicate RAB7 as an oncogene or as an oncosuppressor depending on cellular and tissue contexts, but also on the cancer stage [24–26]. Interestingly, RAB7 has also been involved in the regulation of a number of mitochondrial processes. Indeed, RAB7 is essential during mitophagy to ensure the shaping of isolation membrane around damaged mitochondria [27]. In addition, it was observed that RAB7 has a key role in mitochondria-lysosome contact site formation and tethering, while the action of the RAB7 GTPase-activating protein (GAP) TBC family member 15 (TBC1D15) induces Guanosine Triphosphate (GTP) hydrolysis and it is responsible for contact untethering [28]. The same authors demonstrated that GTP hydrolysis by RAB7 regulates also inter-mitochondrial contact untethering [29]. Notably, in Charcot-Marie Tooth type 2 B (CMT2B), a neuropathy caused by *RAB7* mutations [30–33], these contacts are unphysiologically prolonged [29]. Furthermore, it has been demonstrated that RAB7 is required for mitochondrial antigen presentation guarantying fusion of MDVs with late endosomes, and cytosolic accumulation of MDVs in RAB7 knockout cells was observed [34].

In this work, we have analyzed the endocytic pathway and mitochondrial functions in CDDP-sensitive ovarian cancer cell line, A2780, and in its resistant counterpart, A2780 CIS, a well-established model to investigate the molecular pathways leading to CDDP-resistance in ovarian cancer cells. We provide evidence that, compared to CDDP-sensitive A2780, CDDP-resistant A2780 CIS cells are characterized by mitochondrial and lysosomal defects and an increased production of MDVs that are secreted rather than delivered to lysosomes, implying an impairment of MQC at the basis of CDDP resistance, whose underlying mechanisms are here investigated.

## Methods

### Cells lines and reagents

The A2780 human ovarian cancer cell line (RRID:CVCL_0134) and their cisplatin (CDDP)-resistant counterpart, named A2780 CIS (RRID:CVCL_1942) were acquired by Sigma-Aldrich (St Louis, Missouri, USA) (Catalog Number 93,112,519 and 93,112,517, respectively). From the SKOV3 human ovarian cancer cell lines (RRID: CVCL_0532), the SKOV3 CIS-1 and SKOV3 CIS-2 (CDDP-resistant clones) were obtained as previously described [23]. These cells were grown at 37 °C in an incubator with 5% CO2 in RPMI-1640 medium (Sigma-Aldrich, St Louis, Missouri, USA), supplemented with 10% FBS, 2 mM glutamine, 100 U/mL penicillin, and 10 mg/mL streptomycin (Gibco, ThermoFisher Scientific, Waltham, MA, USA). Cells were regularly evaluated for *Mycoplasma* contamination.

Where indicated, cells were treated with MG132 (20 µM, Sigma-Aldrich), with cycloheximide (CHX) (50 µM, Sigma-Aldrich), carbonyl cyanide *m*-chlorophenylhydrazone (CCCP) (10 µM, Enzo Life Science, NY, USA), GW4869 (20 µM, Cayman Chemical Company, Ann Arbor, Michigan, USA) and with Bafilomycin A1 (400 nM, Santa Cruz Biotechnologies, Santa Cruz, CA, USA).

### Cell viability measurements

Cell viability was measured using the colorimetric MTT (3-(4,5-dimethylthiazol-2yl)-diphenyl tetrazolium bromide) (Sigma-Aldrich, St- Louis, Missouri, USA) assay as previously described [23]. Briefly, cells were plated at a density of 7 × 10^3^ cells/well in 96-well plates and, after 24 h, cells were treated with stepwise-increase concentrations of CDDP for 24, 48, and 72 h. The medium was replaced with 100µL of MTT (1 mg/mL) to each well for 3 h. The formazan crystals produced from metabolizing MTT were dissolved in Dimethyl Sulfoxide (DMSO) (Sigma-Aldrich) and the absorbance, proportional to cell viability, was measured by a Multilabel Plate Reader (Victor X5, PerkinElmer, Waltham, MA, USA) at 570 nm. EC50 was calculated after 72 h as the concentration giving a 50% decrease in the cell number compared to untreated cells using the GraphPad Prism software (Version 8.0, GraphPad, San Diego, CA, USA).

### Seahorse XF flux assay

The Mito Stress Test Kit (Agilent Technology, CA, USA) was used to evaluate the mitochondrial activity and to measure the oxygen consumption rate (OCR). The Glycolytic Rate Assay and the Real-time ATP Rate Assay (Agilent Technology) were used to evaluate the glycolytic activity and ATP production in A2780 and A2780 CIS cells, respectively. Cells were seeded into an 8-well Seahorse XFp Cell Culture Miniplate at the density of 2,5 × 10^4^ cells/well in RPMI complete medium and maintained at 37 °C in an incubator with 5% CO_2_. The next day, the medium was replaced with Seahorse XF RPMI medium supplemented with 1 mM pyruvate, 2 mM glutamine, and 10 mM glucose (Agilent Technology). Cells were incubated for 1 h at 37 °C without CO_2_. The Mito stress test was performed in the Seahorse XFp Extracellular Flux Analyzers (Agilent Technology) and foresees the sequential addition of Oligomycin (1 µM), Carbonyl cyanide-p-trifluoromethoxyphenylhydrazone (FCCP) (1 µM), Rotenone/Antimycin A (0.5 µM). The Glycolytic Rate Assay was performed by sequential addition of Rotenone/Antimycin A (0.5 µM) and 2-deoxy-D-glucose (2-DG) (50 mM) to measure glycolytic rates for basal conditions and compensatory glycolysis following mitochondrial inhibition. The measurement of ATP production was performed by serial injections of Oligomycin (1 µM) and Rotenone/Antimycin A (0.5 µM) to obtain glycolytic and mitochondrial ATP production. For all kits, the instrument performed three measurements for each of the three technical triplicates included in a single plate for each cell line. The data were analyzed by Seahorse Analytics software and normalized on the amount of total proteins/well, determined through the Bicinchoninic acid (BCA) (Thermofisher Scientific) quantification method. Notably, the analysis of mitochondrial activity, using the Mito Stress Test Kit, performed in A2780 cells after 48 h of incubation with A2780 CIS CM and A2780 CIS EV CM (see below 2.12 paragraph), was conducted using Seahorse XF RPMI medium without pyruvate, glutamine, and glucose in order to evaluate only the influence of EVs on mitochondrial activity.

### Extracellular vesicle (EV) purification

For EV collection, cells were washed with Phosphate Buffer (PBS) 1X and incubated with medium containing 10% EV-free FBS for 48 h. The latter was obtained with ultracentrifugation at 120,000 g for 18 h at 4 °C, as indicated by guidelines of the International Society of Extracellular Vesicles (ISEV) [35]. Medium and cells were collected, and EVs were purified by ultracentrifugation and/or immunoisolation. The ultracentrifugation protocol was performed as indicated by ISEV guidelines [35]. Briefly, the conditioned medium was centrifuged at 300 g for 10 min at 4 °C to pellet cells. The supernatant was collected and centrifuged at 16,500 g for 20 min at 4 °C to eliminate apoptotic bodies and cell debris. The supernatant was then filtered through 0.22 μm filters and ultracentrifuged at 110,000 g for 70 min at 4 °C. Pellets were resuspended in PBS1X, and ultracentrifuged at 110,000 g for 70 min at 4 °C. Finally, the pellet containing EVs was resuspended in PBS 1 × [23]. The immunoisolation protocol for EVs was performed using the Exosomes Isolation Kit Pan (Miltenyi Biotec, Germany) following manufacturer’s instructions.

### Transmission electron microscopy (TEM)

TEM analysis of isolated vesicles was performed with a JEOL JEM-1011 transmission electron microscope at 100 kV operating voltage, equipped with a 7.1 megapixel CCD camera (Orius SC1000, Gatan, Pleasanton, CA). TEM image analysis was achieved with Gatan Digital Micrograph™ (DM) software. For sample preparation, a 5 µL drop of a concentrated vesicle suspension was dropped on a Formvar-coated copper grid and then infiltrated with a carboxymethyl dextran solution. The resulting ultrathin polysaccharide layer prevents the vesicle collapse on the dried grids. The protocol was slightly modified from ref [36]. No staining was applied in sample preparation or after deposition on a grid.

### Particle size analysis

The vesicle’s average hydrodynamic diameter as well as the zeta potential measurements (ζ-potential) were performed on a Zetasizer Nano ZSP (Malvern, United Kingdom) equipped with a 10 mW He–Ne laser operating at 633 nm, fixed scattering angle of 173°. The vesicles were diluted with a 0.22 μm-filtered buffer for the analysis. All the measurements were performed at 10 °C.

### Protease protection assay

Protease protection assay was performed as previously described [37]. In this case, the protocol was applied to purified EVs which were incubated with 5 ng/ml proteinase K with or without 1% Triton-X 100 (TX-100) for 10 min at room temperature (r.t.). The reaction was stopped by adding 20 mM phenylmethanesulfonyl fluoride and 2X preheated SDS sample buffer and boiling at 100 °C for 3 min. The samples were subjected to sodium dodecyl sulphate polyacrylamide gel electrophoresis (SDS-PAGE) and immunoblotted with anti-Alix, TSG101, and RAB7 specific antibodies.

### Nuclear fractionation

The preparation of nuclear fraction was performed as previously reported [38]. Briefly, A2780 and A2780 CIS cells were washed with cold PBS, centrifuged, and suspended in ice-cold lysis buffer (50 mM Tris, pH 7.5, 1 mM, 0.1% Triton X-100, 137,5 mM NaCl, 10% glycerol, and 5mM EDTA, supplemented with protease inhibitors) and left in the buffer for 15 min on ice. The nuclei were separated by centrifugation (7000 rpm, 2 min, at 4 °C). The pellet, containing the nuclei, was washed, centrifuged, resuspended in lysis buffer with 0.5% SDS, incubated for 15 min on ice, and centrifuged at 14,000 rpm for 15 min at 4 °C obtaining in the supernatant the nuclear fraction.

### Western blotting

Cells and EVs were lysed in Laemmli Buffer (100 mM Tris–HCl pH 6.8, 4% SDS, 20% glycerol, and 0.2% blue bromophenol) and then quantified using the BCA assay (Thermofisher Scientific) or by Stain-free gels (Bio-Rad, Hercules, CA, USA). Proteins were separated by (SDS-PAGE) and subsequently electroblotted onto polyvinylidenefluoride (PVDF) Immobilon-P membranes (Millipore, Billerica, MA, USA) as previously reported [39]. Primary antibodies were incubated overnight and anti-mouse and anti-rabbit peroxidase-conjugated secondary antibodies for 1 h at r.t. The complete list of primary and secondary antibodies is shown in Table S1. The purity of EVs was ascertained according to the ISEV guidelines [35]. The presence of the cytosolic protein Alix, Tumor Susceptibility gene 101 (TSG101), and three tetraspanins CD63, CD9, and CD81 (positive controls), and the absence of the non-EV component Ribosomal Protein S6 (RPS6) (negative control) were verified. Images were acquired by the ChemiDoc MP Imaging System and analyzed by Image Lab TM software version 6.0.1 (Bio-Rad Laboratories). Protein expression levels were quantified by densitometry normalizing against the housekeeping bands or the total proteins.

### DNA extraction from EVs and mitochondrial DNA amplification

DNA extraction from EVs isolated by ultracentrifugation was performed as reported previously [40]. Briefly, EVs were incubated with 1U of Baseline-ZERO™DNase0 (Epicentre, Madison, Wisconsin) for 30 min at 37°C, in order to eliminate endogenous ss- and ds-DNA. Enzymes were heat inactivated by an incubation of 10 minutes at 65°C. Whole EV DNA was isolated using phenol/chloroform (ThermoFisher Scientific) extraction. One volume of phenol pH 8 was added to each sample of cell pellets and EVs resuspended in 1X PBS and centrifuged at 12000 rpm for 5’ at r.t. The upper phase, containing the DNA, was transferred to a new tube where 1 volume of chloroform was added. Samples were centrifuged at 12,000 rpm for 5’ at r.t. The upper phase was transferred to a new tube and 1/10 of the volume of sodium acetate 3 M pH 6.5 and 1 µl of glycogen were added and vortexed. Subsequently, 2.5 volumes of 100% cold EtOH was added, and mixed and the sample was incubated at -20 °C overnight. The day after, samples were centrifuged at 13,000 rpm for 15 min at 4 °C. The supernatant was discarded, and the DNA pellet was washed with 200 µl of 70% cold EtOH and centrifuged at 13,000 rpm for 10 min at 4 °C. The DNA pellet was then air dried and resuspended in 50 µl of Tris-HCl 10mM pH8.5. DNA concentration was measured by loading 1 µl of DNA on a Thermo Scientific NanoDrop™ 1000 Spectrophotometer. DNA was stored at -20 °C until further analyzed. Total DNA (10 ng) was used for mitochondrial DNA (mtDNA) amplification. PCR with MitoALL resequencing kit (Applera) was used as previously reported [41] and selecting only the following amplicon numbers: 12, 13, and 20 to amplify *MT-ND6*; 19 and 20 for *MT-RNR1*; 27 for *MT-COX-1*; 28 and 40 to amply *MT-COX-2*); finally, amplicon 41 to amplify part of *MT-ND3* and *MT-ND4L* genes. For the amplification reaction, AmpliTaq DNA Polymerase (Applied Biosystems) and T100 Thermal Cycler (Bio-Rad) were used.

### Live and confocal microscopy

Cells were seeded into microscopy chambers (8 well µ-slide, Ibidi GmBh, Martinsried, Germany) and after 24 h incubated with MitoTracker Red CM-H_2_XROS (50 nM), LysoTracker Green DND-26, MitoTracker Green FM (50 nM), and/or LysoTracker Red DND-99 (1 µM) (ThermoFisher Scientific) as previously reported [23, 42]. Briefly, cells were incubated with dyes for 45 min at 37 °C in medium without FBS, washed three times in PBS, and finally incubated with L-15 medium (Leibowitz medium without phenol red, Gibco, ThermoFisher).

EVs were labeled with DiI (1,10 -dioctadecyl-3,3,3’,3’-tetramethylindocarbocyanine perchlorate; λ_Ex_/λ_Em_ = 549/565 nm) as previously described [43]. Briefly, after purification with the ultracentrifugation method, EVs were incubated with DiI (500nM) for 45 min. Subsequently, DiI-stained EVs were washed twice with PBS using ultracentrifugation (110,000 g for 70 min), finally resuspended in RPMI EV-free, and added to cells for 48 h.

Cells were seeded on 11 mm round glass coverslips in 24 well plates to achieve 70–80% confluency and in microscopy chambers (8 well µ-slide, Ibidi GmBh, Martinsried, Germany). After 24 h, cells were treated with medium containing DiI-stained EVs. For immunofluorescence, cells were fixed in 3% paraformaldehyde for 20 min, permeabilized with PBS -Saponin 0.25% buffer for 10 min, washed in PBS-Saponin 0.1%, and incubated with the primary antibody in PBS-Saponin 0.1% for 20 min. After 3 washes with PBS-Saponin 0.1%, samples were incubated for 20 min with the secondary antibody in PBS-Saponin 0.1%, washed three times with PBS-Saponin 0.1%, and finally rinsed in PBS 1X. Coverslips were then mounted on a drop of Mowiol (Calbiochem-Novabiochem Corporation, La Jolla, CA, USA). Fluorescence images were captured using a confocal laser scanning microscope (CLSM) (Zeiss, LSM 700, Germany). The images were acquired using ZEN Black Edition 2011 software (Zeiss, Jena, Germany) and fluorescence intensity was quantified using ImageJ software (Version 1.5Oi, Bethesda, MD, USA).

### DQBSA (Self-Quenched BODIPY dye conjugates of bovine serum albumin) assay

Cells were seeded on 11 mm round glass coverslips and incubated in the presence of Green DQ-BSA (50 µg/mL) (ThermoFisher Scientific) for 24 h at 37 °C with the full medium in a humidified atmosphere of 5% CO2. Subsequently, cells were fixed in methanol and analyzed with LSM 700 confocal microscope (Zeiss). Images were taken with a Plan-Apochromat 63.0 × 1.40 oil-immersion objective DIC M27 and the pinhole aperture was set to 1 Airy unit. Images were acquired using ZEN Black Edition 2011 acquisition software (Zeiss, Germany).

### Quantitative real-time PCR

RNA extraction and quantitative Real-Time PCR (qRT-PCR) were performed as described previously [39]. The primers used are reported in Table S1.

### Transfection

A2780 CIS cells were transiently transfected, using Metafectene Pro from Biontex (Martinsried, Germany) as previously described [23], with plasmid pcDNA3.1 vector (Invitrogen, V79020) encoding 2xHA (mock) and 2xHA-RAB7A wild type [44], with GFP-RAB7A wild-type and with GFP-Microtubule Associated Protein 1, Light Chain 3 (LC3), kindly gifted by Dr. M. Chiariello (Core Research Laboratory-Siena, Institute for Cancer Research and Prevention; Institute of Clinical Physiology, National Research Council, CNR). For the growth curve with CCDP, after 3 h of transfection, the medium was replaced and the cells were treated with stepwise-increase concentrations of CDDP in RPMI 12,5% FBS for 48 h.

### EV transfer using conditioned medium from A2780 CIS and A2780 cells

A2780 CIS and A2780 cells were seeded at 5 × 10^6^ cells/p150 Petri dishes (Corning) in EV-free RPMI and grown for 96 h. To obtain A2780 CIS conditioned medium (CM), the medium from A2780 CIS cells was first centrifuged at 300 g for 10 min, subsequently at 16,500 g for 20 min, and finally filtered through 0.22 μm filters. To obtain the EVs from A2780 CIS and A2780, the CMs from A2780 CIS and A2780 cells were ultracentrifuged and both supernatant and pellet were collected (see paragraph 2.4). For A2780 CIS cells, the supernatant represented the CM deprived of EVs (A2780 CIS CM w/o EVs), while the EV pellet was resuspended in 5 ml of RPMI containing 10% of EV-free FBS (A2780 CIS EVs). Similarly, the EV pellet from the ultracentrifuged A2780 cell medium was resuspended in 5 ml of RPMI containing 10% of EV-free FBS (A2780 EVs). In order to obtain a conditioned RPMI medium deprived of EVs in an alternative way, A2780 CIS cells were treated for 48 h with 20µM of GW4869 (Cayman Chemical Company, Ann Arbor, Michigan, USA), an inhibitor of EV secretion, and then the medium was prepared as above indicated. All centrifugations and ultracentrifugations were performed at 4 °C. A2780 cells were then grown in CM for 96 h before analysis.

In some experiments A2780 cells were seeded at 5 × 10^6^ cells/p150 Petri dishes (Corning) in EV-free RPMI, grown for 96 h, and treated with CCCP (10 µM) for 18 h. Untreated A2780 cells were used as control. To obtain EVs from untreated and CCCP-treated A2780, the CM was prepared as above. The media were ultracentrifuged, the pellets were collected (see paragraph 2.4), and finally resuspended in 5 ml of RPMI containing 10% EV-free FBS (A2780 EVs and A2780 CCCP EVs CM). Then, A2780 cells were subjected to treatment with stepwise concentrations of CDDP dissolved in the two CM media for 72 h before analysis.

### 2′,7′-Dichlorodihydrofluorescein diacetate staining

2′,7′-Dichlorodihydrofluorescein diacetate (DCFH-DA) staining was performed to determine total cellular reactive oxygen species (ROS) that underlie oxidative stress. A2780 and A2780 CIS cells were seeded (8 × 10^4^ per well) in a 24-well plate and treated with DMSO or with 100 µM H_2_O_2_ in DMSO for 4 h. Total ROS were then detected by the DCFH-DA (Thermo Fisher Scientific) assay. The drug was added to each well at a concentration of 10 µM and incubated at 37 °C for 30 min. Then, cells were washed three times with PBS, lysed in 100 µL of RIPA (radioimmunoprecipitation assay) buffer for 5 min on ice, and centrifuged at 10,000 rpm for 10 min at 4°C. 90 µL of the supernatant were transferred to a 96-well plate and fluorescence at 490 nm was read using a microplate reader (Victor X5, Perkin Elmer). The remaining part of the lysate was used to measure protein concentration by Bradford assay and to normalize fluorescence intensities on the protein concentration of each sample.

### Statistical analysis

Colocalization rates were determined using the ZEN 2011 software (Carl Zeiss, Oberkochen, Germany) as the weighted colocalization coefficient, as previously described [45, 46]. Fluorescence intensity was evaluated by quantifying Correct Total Cell Fluorescence (CTCF), while DQ-BSA puncta in cells and the number and size of acid compartments were determined through Analyze Particle tool of ImageJ software [45–47]. Measures were obtained by analyzing at least 50 cells/sample for at least three independent experiments. Results are represented as mean ± standard error (SEM). Statistical significance was determined for all experiments through Student’s t-test for unpaired data (*p ≤ 0.05, ** *p* ≤ 0.01, and *** *p* ≤ 0.001).

## Results

### CDDP-resistant A2780 CIS ovarian cancer cells are characterized by dysfunctional lysosomes

**Fig. 1:**
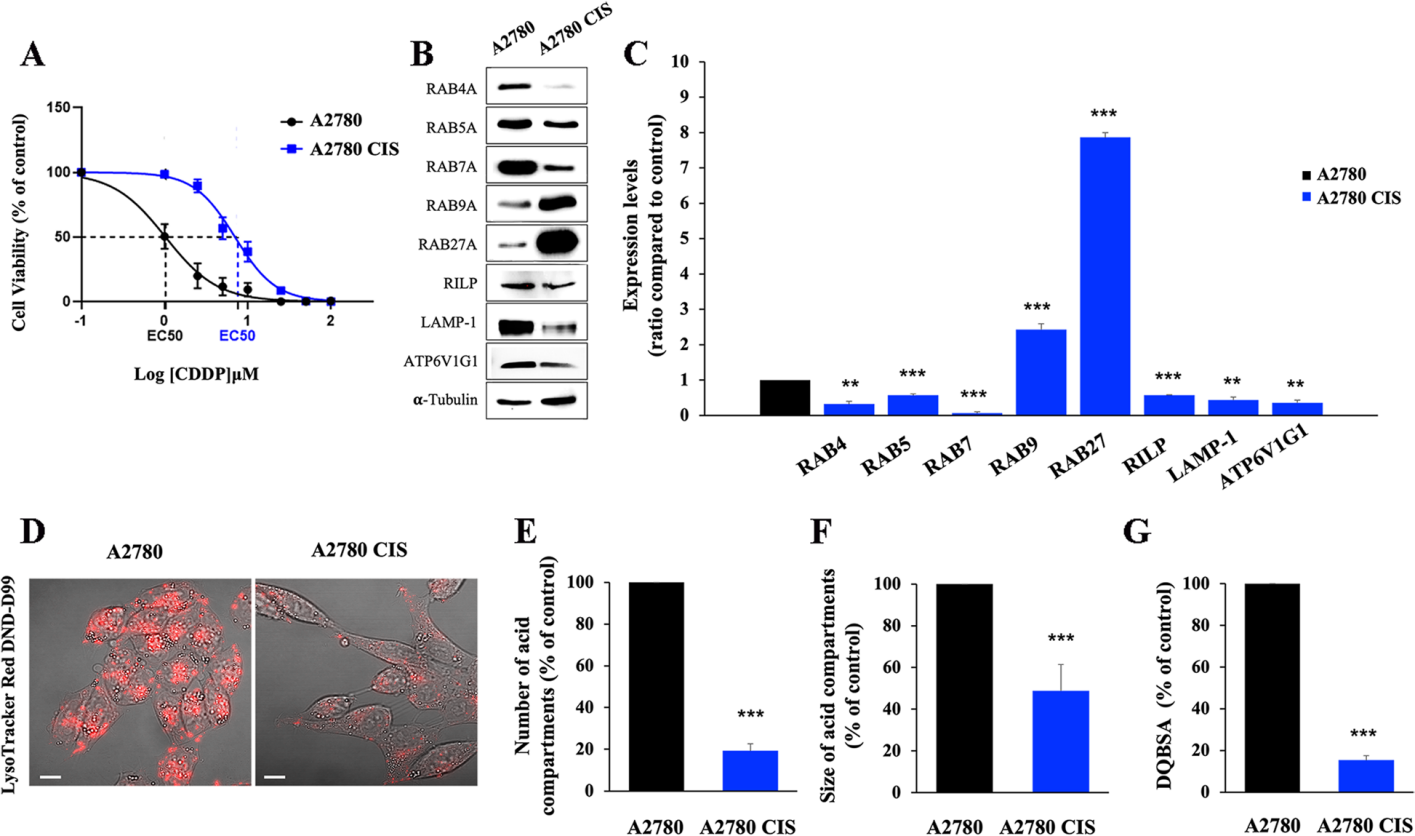
Impairment of the endocytic pathway in chemoresistant cells. **A** EC50 was determined in A2780 and in A2780 CIS cells after 72 h of treatment with CDDP using the MTT(3-(4,5-dimethylthiazol-2-yl)-2,5-diphenol tetrazolium bromide) assay. **B**,** C** Relative protein abundance of RAB4, RAB5A, RAB7, RAB9, RAB27, RILP, LAMP-1, and ATP6V1G1 was assessed by Western blot analysis and quantified by densitometry normalizing against α-Tubulin. **D** Late endocytic acid compartments were live stained with LysoTracker DND-99 dye and images were acquired with confocal microscopy. **E**,** F** The number and size of LysoTracker-positive organelles were determined by ImageJ software. **G** Quantification of Green DQBSA puncta per cell was determined by ImageJ software. Data represent the mean ± SEM of at least three independent experiments. * *p* < 0.05; ** *p* < 0.01; ***p < 0.001

In recent years the importance of the interaction between organelles in the regulation of cellular homeostasis has emerged, and the role of contact sites between them and, in particular, between mitochondria and lysosomes, has subverted the old conception of cellular compartments as self-sufficient. Moreover, recent evidence suggests that mitochondria-lysosome crosstalk is mainly regulated by RAB7 [28, 29]. The available evidence suggests that this crosstalk may modulate sensitivity to CDDP and therefore we set out to contextually analyze the endocytic pathway and mitochondrial functions in CDDP-sensitive ovarian cancer cell line, A2780, and in its resistant counterpart, A2780 CIS (Fig. 1A). Indeed, on one hand, we previously shown that RAB7 plays a role in chemoresistance regulation, as modulation of RAB7 expression is able to influence CDDP response, and that impairment of the late endocytic pathway is a feature of several CDDP-resistant cell lines [23]. On the other hand, there is evidence that in ovarian cancer cells mitochondrial DNA (mtDNA) is critically affected by CDDP, and that sensitivity to CDDP of these cells is correlated with higher mitochondrial content and higher levels of mitochondrial ROS [48]. Moreover, it may be noted that A2780 CIS cells harbor a heterozygous missense mutation affecting p53 gene resulting in the K351N substitution [49]. In addition to affect the stability of p53 tetramers and p53 transcriptional activity, this mutation has been shown to impair ubiquitination of p53, and CDDP-induced translocation of p53 to mitochondria [49].

We started to analyze the levels of expression of RAB7 and other proteins involved in the endocytic pathway, such as RAB4A, RAB5A, RAB9A, RAB27A, the Rab-interacting lysosomal protein (RILP), the Lysosomal Associated Membrane Protein 1 (LAMP-1), and the V1G1 subunit of the vacuolar ATPase H + Transporting (ATP6V1G1) (Fig. 1B) comparing chemoresistant and chemosensitive ovarian cancer cells. RAB4A is involved in the regulation of constitutive recycling cargo exit from early sorting endosomes directly back to the plasma membrane as well as to the recycling compartment, while RAB5A regulates homotypic fusion and motility of early sorting endosomes [50]. RAB7, through interaction with its effector RILP, is able to control late endocytic trafficking and multivesicular bodies (MVBs) formation [51–53]. RILP regulates the endocytic pH by controlling the assembly and activity of the V-ATPase on RAB7A-positive organelles through interaction with the ATP6V1G1 subunit [42, 54, 55], while LAMP-1 is a late endocytic marker protein, and its expression is frequently used as an indicator of lysosomal abundance. RAB9A is a small GTPase, segregated in RAB7-distinct domains within late endosome membranes [56], responsible for mannose-6-phosphate receptor transport from late endosome to the trans-Golgi apparatus [57]. RAB27A regulates exosome secretion being essential for docking multivesicular bodies (MVBs) to the plasma membrane [58].

We found that RAB4A, RAB5A, RAB7A, RILP, LAMP-1, and ATP6V1G1 proteins were significantly downregulated in A2780 CIS chemoresistant cells (Fig. 1B, C) allowing to hypothesize the presence in these cells of a dysfunctional endocytic pathway. In contrast, we found a significant increase of 2.5 and 8 fold of RAB9 and RAB27 expression, respectively, suggesting an increased production of EVs (Fig. 1B, C). We confirmed late endocytic dysfunction in these cells by performing a live-cell imaging assay using the LysoTracker DND-99 probe. As shown in Fig. 1D, CDDP resistant cells are characterized by a very strong decrease of the fluorescent signal compared to their sensitive counterpart and quantification confirmed a significant decrease in the number and size of acidic compartments (namely late endosomes and lysosomes) (Fig. 1E, F). Furthermore, using the DQBSA assay to measure lysosomal proteolytic activity [9], we observed a strong reduction of DQBSA fluorescent puncta in A2780 CIS cells compared to A2780, indicating a decrease of lysosomal activity in chemoresistant cells (Fig. 1G). All these findings were indicative of impairment of the late endosome and lysosome compartments in A2780 CIS cells.

### CDDP-resistant A2780 CIS ovarian cancer cells are characterized by deficit in mitochondrial bioenergetics

**Fig. 2:**
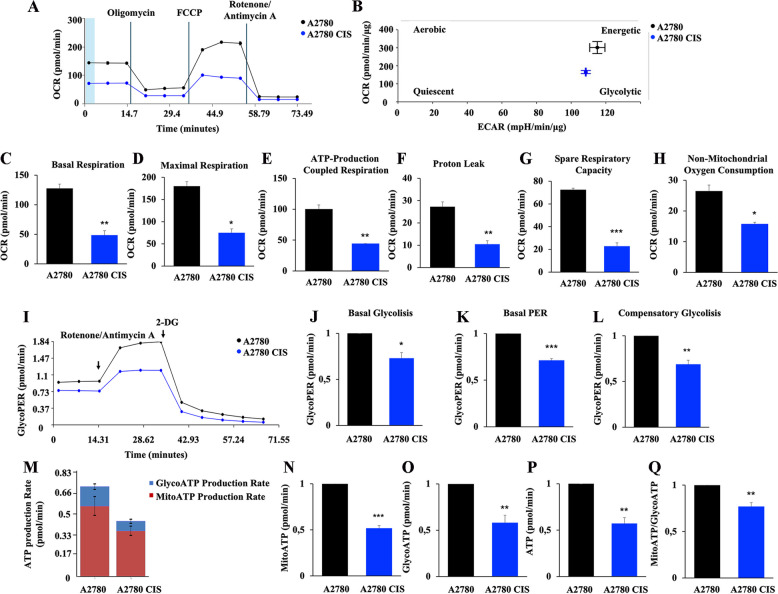
Defective mitochondrial phenotype in chemoresistant cells. **A** Oxygen rate consumption (OCR) was determined in A2780 and A2780 CIS cells with Seahorse Mito stress kit assay. **B** OCR and Extracellular Acidification Rate (ECAR) obtained with Seahorse instruments allowed to determine the energetic map of A2780 and A2780 CIS cell lines. **C**-**H** Basal respiration, maximal respiration, ATP-production coupled respiration, proton leak, spare respiration capacity and non-mitochondrial oxygen consumption were determined by Seahorse data elaboration. **I** Glycolytic Proton Efflux Rate (GlycoPER) was determined with the Seahorse Glycolytic Rate Assay kit. **J**-**L** Basal Glycolysis, Basal PER, and Compensatory Glycolysis were determined by Seahorse data elaboration. **M** ATP production Rate was measured with Seahorse instrument and **N-Q** subsequent elaboration data have allowed determining ATP produced by mitochondria (MitoATP), by the glycolysis route (GlycoATP), total ATP and MitoATP/GlycoATP ratio. Data represent the mean ± SEM of at least three independent experiments. * *p* < 0.05; ** *p* < 0.01; ***p < 0.001

Then, we analyzed mitochondrial functions in both cell lines using the Seahorse Mito Stress Kit assay (Fig. 2A). Interestingly, we found that in A2780 CIS cells the oxygen consumption rate (OCR) was decreased compared to A2780 chemosensitive cells. Moreover, beyond the measurement of mitochondrial oxidative phosphorylation (OXPHOS) on the basis of the oxygen consumption rate (OCR), the Seahorse instrument is able to measure indirectly glycolysis by analyzing the extracellular acidification rate (ECAR). The energetic map analysis (Fig. 2B), revealed that both cell lines are not quiescent, but exhibit a more glycolytic than aerobic energetic metabolism, as expected from cancer cells. In addition, all analyzed parameters, such as basal and maximal respiration, ATP production, proton leak, spare respiratory capacity, and non-mitochondrial OCR were downregulated in the A2780 CIS cell line (Fig. 2C-H). In particular, the more informative parameter is the proton leak (Fig. 2F) that, being indicative of remaining basal respiration not coupled to ATP production, can be a signal of mitochondrial damage, while spare respiratory capacity (Fig. 2G) indicates the capability of the cell to respond to an energetic demand and thus how closely the cell is next to respire to its theoretical maximum being an indicator of cell fitness or flexibility. Furthermore, the glycolytic activity (Fig. 2I) was measured using the Seahorse Glycolytic Rate assay. We found a reduction of glycolytic proton efflux rate (glycoPER), which is the number of protons exported by cells into the assay medium derived by glycolysis, that highly correlates with lactate production. Thus, basal glycolysis and basal PER are significantly reduced in A2780 CIS cells (Fig. 2J, K). Similarly, compensatory glycolysis, which is the rate of glycolysis in the cells consequent to OXPHOS inhibition, which indicates the capacity of the cells to respond to energetic demand through glycolysis, was found significantly decreased in chemoresistant cells (Fig. 2L). Altogether these findings were indicative of an impairment of energy metabolism in A2780 CIS cells, and indeed, when we analyzed ATP production using the Real-time ATP Rate assay, we observed a significant decrease in the production of ATP, both mitochondrial and glycolytic, in chemoresistant A2780 CIS cells (Fig. 2M-P). Interestingly, albeit the mitochondrial/glycolytic ATP ratio is decreased in chemoresistant cells, both cell lines are characterized by prevalent energy production by glycolytic metabolism (Fig. 2M) as expected by cancer cells.

**Fig. 3:**
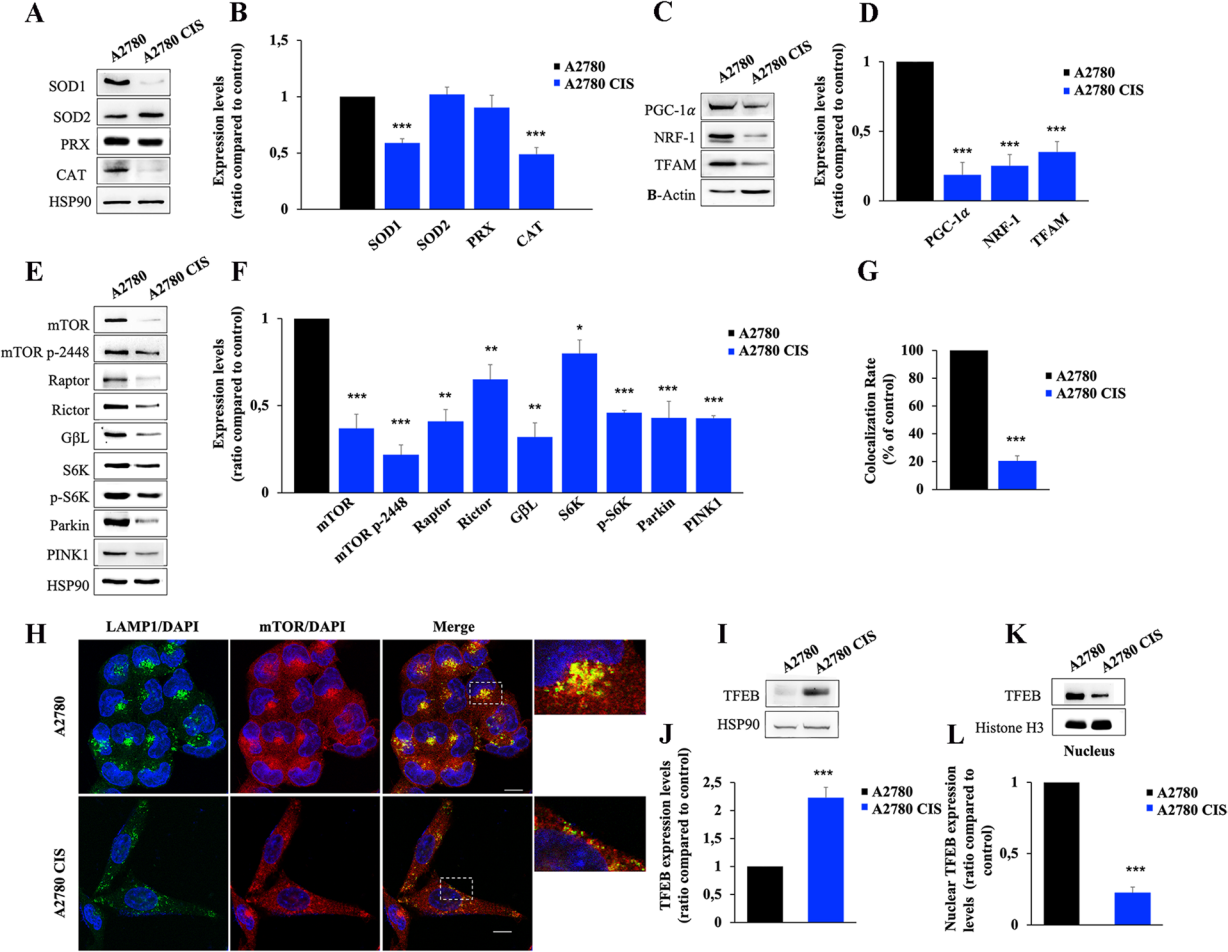
Alterations of mitochondrial and lysosomal biogenesis regulators characterize chemoresistant cells. **A**, **B** Relative protein abundance of SOD1, SOD2, PRX, and CAT was assessed by Western blot analysis and quantified by densitometry normalizing against HSP90. **C**, **D** Relative protein abundance of PGC-1α, NRF-1, and TFAM was assessed by western blot analysis and quantified by densitometry normalizing against β-Actin. **E**, **F** Relative protein abundance of mTOR, mTOR p-2448, Raptor, Rictor, GβL, S6K, p-S6K, Parkin, and PINK1 was assessed by Western blot analysis and quantified by densitometry normalizing against HSP90. **G**, **H** Colocalization rates were determined for A2780 and A2780 CIS cells immunostained with anti-mTOR (red) and anti-LAMP-1 (green) antibodies. Nuclei were stained with DAPI (blue). White boxes indicated zoomed areas on the right. Scale bar = 10 μm. **I**, **J** The total TFEB abundance was determined by Western Blot analysis and quantified by densitometry normalizing against HSP90 using Image Lab software (Bio-Rad). **K**, **L** Relative protein abundance of the nuclear fraction of A2780 and A2780 CIS subjected to Western blotting using anti-TFEB, and anti-Histone H3, used as the loading control, was determined. For densitometry Image Lab software (Bio-Rad) was used. Data represent the mean ± SEM of at least three independent experiments. * *p* < 0.05; ** *p* < 0.01; ***p < 0.001

To understand the severity of mitochondrial damage, we analyzed the expression levels of mitochondrial reactive oxygen species (ROS) scavenger proteins [59]. We analyzed superoxide dismutase 1 (SOD1), mitochondrial SOD2, peroxiredoxin (PRX), and catalase expression in the two cell lines (Fig. 3A). We found that SOD1 and catalase were significantly downregulated, while SOD2 and PRX enzymes were unchanged in chemoresistant cells (Fig. 3B). Furthermore, the quantification of ROS production by spectrophotometric determination of dichlorodihydrofluorescein diacetate (DCFH-DA) reduction indicated that ROS did not increase in chemoresistant cells (Supplementary Fig. S1). These data indicate the absence of increased oxidative stress in A2780 CIS, possibly coherent with a reduced OCR in these cells. Indeed, mitochondria of A2780 CIS cells are less functional and produce less ROS, which are scavenged from sufficient levels of mitochondrial SOD2 and PRX.

### The TFEB-mTOR pathway overseeing lysosomal and mitochondrial biogenesis and turnover is down-regulated in CDDP-resistant A2780 CIS ovarian cancer cells

To understand if the decrease of OCR and ATP production was due to changes in regulatory pathways overseeing mitochondrial biogenesis, activity and turnover, we analyzed expression levels of peroxisome-proliferator-activated receptor coactivator (PGC)-1α, which is the master regulator of mitochondrial biogenesis and mitochondrial respiration, of nuclear respiratory factor 1 (NRF-1) and of mitochondrial transcription factor A (TFAM) [60, 61] (Fig. 3C). Indeed, PGC-1α co-activates NRF-1, which stimulates the expression of a large number of nuclear genes involved in mitochondrial respiration and in mitochondrial DNA (mtDNA) replication and transcription. One of these is the gene coding for TFAM, a protein that regulates both mtDNA transcription and mtDNA replication [62, 63]. We found that PGC-1α, NRF-1, and TFAM were downregulated in CDDP resistant cells compared to control chemosensitive cells (Fig. 3C, D).

Furthermore, it is known that the mammalian target of rapamycin (mTOR) interacts with PGC-1α to regulate mitochondrial biogenesis and mitochondrial oxidative function [40]. Analysis of the mTOR pathway displayed that proteins of this route are all downregulated in chemoresistant cells (Fig. 3E, F). Notably, also basal levels of Parkin and PTEN-induced kinase1 (PINK1) were significantly decreased in A2780 CIS cells, allowing to suppose also dysfunction of mitophagy as a mechanism of mitochondrial quality check (MQC) (Fig. 3E, F). Moreover, mTOR is localized on lysosomes where it interacts with RAB7 [26, 64]. Depletion of RAB7 reduces the overactivation of mTOR and its activation occurs when localized on lysosomes [65]. In light of these premises, we investigated the co-localization rate between mTOR and LAMP-1 in chemosensitive and chemoresistant cells by immunostaining and we observed a significant decrease of mTOR localization on lysosomes in A2780 CIS cells (Fig. 3G, H).

It was shown that mTOR signaling and lysosomal tethering requires Transcription Factor EB (TFEB) transcriptional activity [66]. TFEB is a master transcriptional regulator of cell catabolism, globally controlling the expression of genes that regulate lysosomal and autophagosomal biogenesis [67]. Recently, it was identified a novel role of TFEB in the promotion of the endocytic pathway during starvation to sustain lysosomal function and autophagy [68]. Notably, recent work demonstrated also that TFEB induces mitochondrial biogenesis via PGC-1α [69]. Considering these data, we analyzed the total and nuclear amount of TFEB (Fig. 3I-L) to establish nuclear translocation in ovarian cancer cell lines and, coherently with the reduced expression of mTOR pathway proteins, we found a significant increase in the total amount of TFEB (Fig. 3I, J), but also reduction of nuclear abundance, and hence translocation, in A2780 CIS cells (Fig. 3K, L).

### CDDP-resistant A2780 CIS ovarian cancer cells are characterized by impairment of MQC

The above results demonstrate that the TFEB-mTOR pathway was down-regulated in CDDP-resistant A2780 CIS compared to CDDP-sensitive A2780 cell line. Down-regulation of this pathway may be responsible for dysfunctional MQC mechanism. In fact, the mTOR protein also plays a crucial role in regulating autophagy in concert with TFEB, whose role in MQC was also demonstrated [69, 70]. TFEB activates and is activated by the PINK1-Parkin signaling pathway in a virtuous cycle to induce mitophagy activation and lysosomal biogenesis, enhancing the ability to degrade mitochondria [69].

**Fig. 4:**
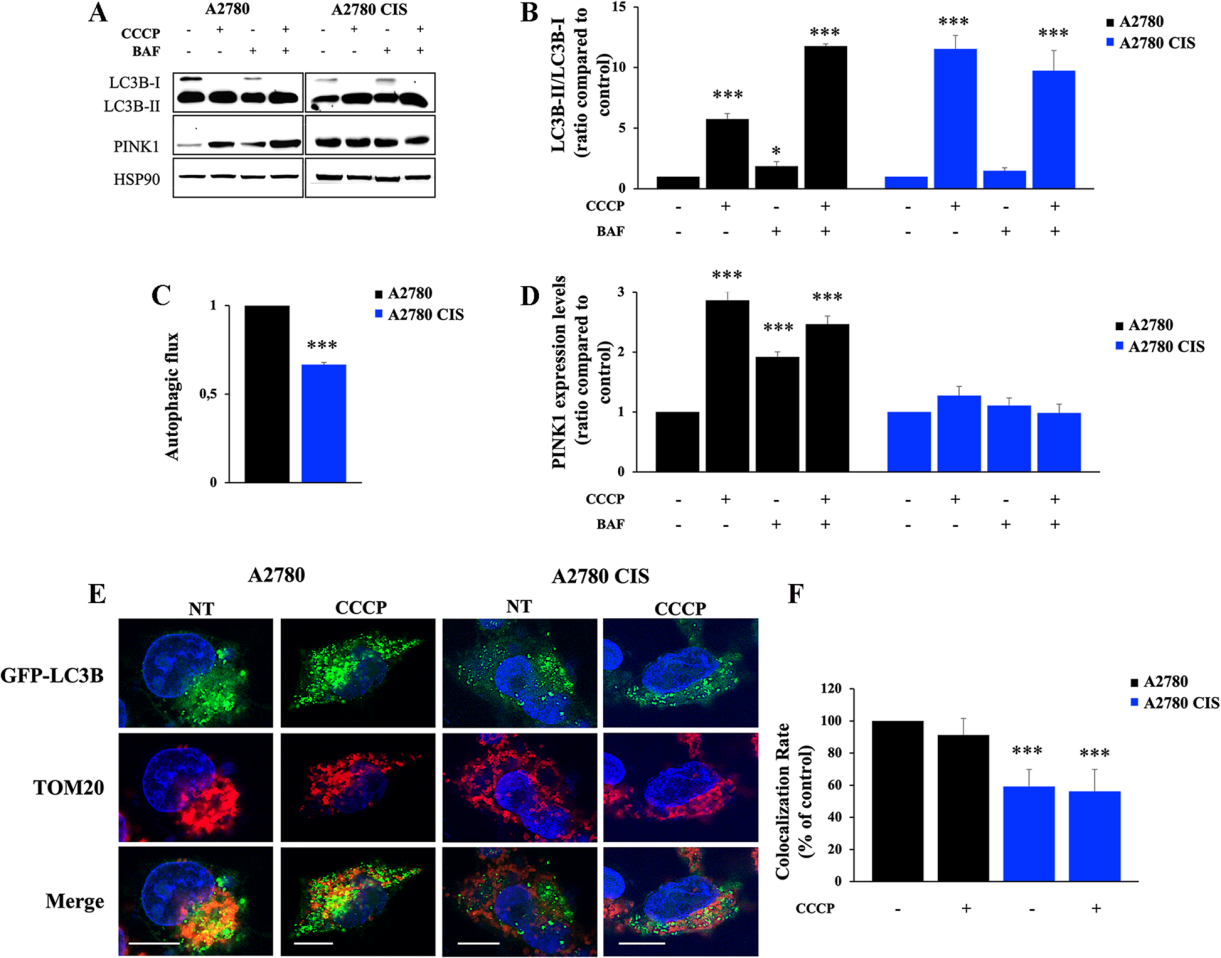
Analysis of mitophagy in A2780 and A2780 CIS cell lines. **A** A2780 and A2780 CIS cells were incubated in full medium, with 10 µM CCCP for 18 h and/or with 400 nM Bafilomycin A1 (BAF) for 3 h. Western blot analysis was performed on cellular lysates using antibodies against LC3B and PINK1. HSP90 was used as a loading control. Densitometric analysis of immunoblot shown in (**B**) and (**D**) was performed using Image Lab software (Bio-Rad). **C** The autophagic flux was calculated as the ratio of normalized LC3BII between BAF and untreated cells of the same sample. **E** A2780 and A2780 CIS expressing GFP-LC3 (green) were treated with CCCP and subjected to immunofluorescence analysis using an antibody against TOM20 (red). Nuclei were stained with DAPI (blue). Scale bar = 10 μm. **F** The colocalization rates of mitochondria (TOM20, red) and autophagosomes (GFP-LC3, green) were determined with ZEN Black Edition 2011 acquisition software (Zeiss, Germany). Data represent the mean ± SEM of at least three independent experiments. * *p* < 0.05; ** *p* < 0.01; ***p < 0.001

As we found downregulation of PINK1 and Parkin proteins in chemoresistant cells (Fig. 3E, F), we hypothesized that in these cells mitophagic degradation was dysfunctional. In light of this, we treated A2780 and A2780 CIS cells with Bafilomycin A1, which inhibits the vacuolar type H+ -translocation ATPase (V-ATPase), and/or with CCCP, which induces (i) depolarization of mitochondria, (ii) PINK1 accumulation, (iii) recruitment of Parkin and (iv) mitophagy concretization. Then, we analyzed the abundance of the autophagosomal marker LC3B, of PINK1 and we evaluated the autophagic flux (Fig. 4A-D) [71]. As expected, we observed a significant increase of LC3B-II in A2780 cells in all tested conditions compared to untreated cells and in A2780 CIS cells treated with CCCP only or with CCCP and Bafilomycin. In contrast, A2780 CIS cells treated only with Bafilomycin A1 didn’t show an increase of LC3B-II compared to untreated cells (Fig. 4A, B). In fact, the analysis of autophagic flux indicated an inhibition in these cells (Fig. 4C). Moreover, deepening the gaze on mitophagic markers, accumulation of PINK1 upon the different treatments occurred only in chemosensitive cells (Fig. 4A, D). To confirm the impairment of this MCQ mechanism, we transfected GFP-LC3B and treated both cell lines with CCCP to analyze co-localization between autophagosomes and mitochondria (Fig. 4E, F). After immunostaining of the mitochondrial import receptor subunit 20 (TOM20), we observed reduced co-localization in untreated and CCCP-treated A2780 CIS cells (Fig. 4F). These results indicate that CDDP-resistant A2780 CIS ovarian cancer cells are characterized also by impairment of autophagy, and, in particular, of mitophagy resulting in dysfunctional MQC, mitochondrial turnover, and lysosome biogenesis.

### CDDP-resistant A2780 CIS cells are characterized by increased secretion of RAB7-containing EVs

**Fig. 5:**
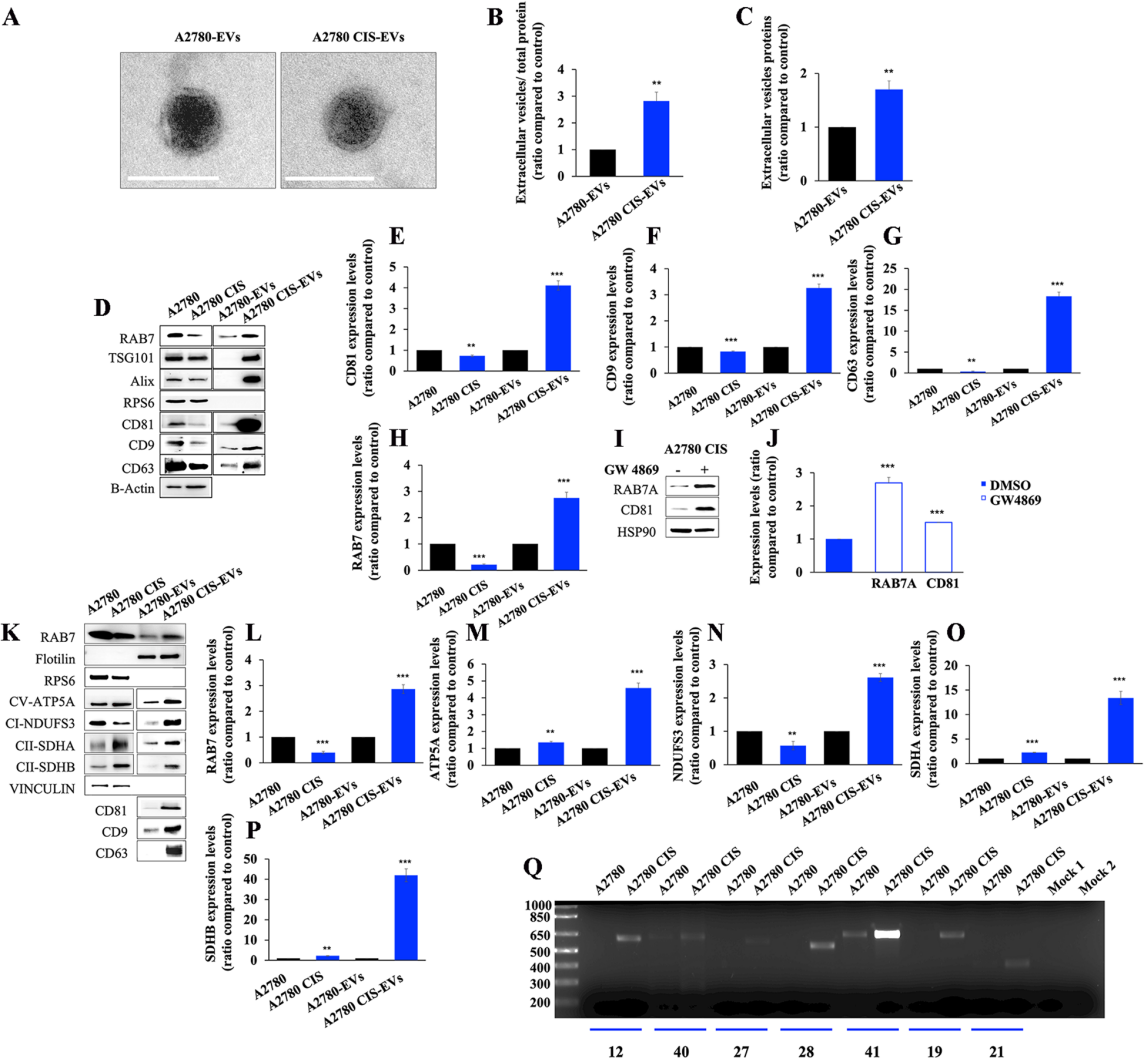
Analysis of EV content. **A** EV morphology was determined by Transmission Electron Microscopy (TEM). Scale bar = 100 nm. **B**, **C** The total amount of extracellular proteins released by A2780 and A2780 CIS cells normalized on total cell proteins and without normalization. **D** EVs were purified by ultracentrifugation and RAB7 content in cell lysates and EVs were evaluated by Western blot analysis. The purity of EVs was ascertained according to the ISEV guidelines by looking at different negative and positive markers as indicated. **E**-**H** Expression levels of CD81, CD9, CD63, AND RAB7 were determined in cellular lysates and in EVs by densitometry normalizing against β-Actin. **I**-**J** A2780 CIS were treated with GW4869 EV secretion inhibitor and intracellular levels of RAB7 and CD81 (as a positive control of inhibition) were measured by western blotting analysis normalizing against HSP90. **K**-**P** EVs were purified by immunoisolation. Positive and negative controls were used to verify the purity of EVs. Mitochondrial protein expression was evaluated in EVs by Western blot and the relative abundance of RAB7, ATP5A, NDUFS3, SDHA, and SDHB was determined through densitometric analysis normalizing against vinculin. **Q** Total DNA was extracted from A2780 cells and A2780 CIS EVs. MitoALL resequencing kit was used to analyze mitochondrial DNA (mtDNA) contained in EVs. Mock 1 and Mock 2 represent two conditions of PCR purity control in which PCR mix with amplicon 19 and 21 primers were used as controls. Data in graphs represent the mean ± SEM of at least three independent experiments. * *p* < 0.05; ** *p* < 0.01; ***p < 0.001

We wondered whether the increased secretion of EVs observed in CDDP-resistant versus CDDP-sensitive cells [23] could be a cause or effect of MQC dysfunction. To address these issues, a molecular characterization of EVs from CDDP-resistant and CDDP-sensitive cells was performed. We purified EVs from the medium of A2780 and A2780 CIS cell lines through the ultracentrifugation method and, as indicated by the International Society of Extracellular Vesicles (ISEV), we verified morphology and size of these vesicles through Transmission Electron Microscopy (TEM) and Dynamic Light Scattering (DLS) (Fig. 5A and S2).

The morphological analysis performed by TEM on about 130 EVs for each cell line (Fig. 5A and S2) revealed a quasi-spherical shape for the vesicles, with minor differences between the two cell lines, and the purity of EV samples, as shown in supplementary data (Figure S2).

To determine the amount of secreted EVs, we quantified by Bicinchoninic acid (BCA) assay the purified fractions of EVs. By normalizing the data on total cell proteins, we found that A2780 CIS cells were able to secrete 3-fold more EVs compared to the A2780 sensitive cell line (Fig. 5B). Similarly, by analyzing the absolute amount of EV proteins, we found that A2780 CIS cells were able to secrete 1.7-fold more EVs compared to the A2780 sensitive cell line (Fig. 5C). Analysis of EV markers by western blot revealed that A2780 CIS cells were characterized by an increase of CD63, CD9, and CD81 in EVs compared to A2780 cells, accompanied by a significant reduction of the same tetraspanins inside the cells (Fig. 5D, E, F, G). Interestingly, albeit expression of RAB7 was strongly reduced in A2780 CIS cells, it was instead increased in EVs from these cells compared to the ones from CDDP sensitive A2780 cell line (Fig. 5D, H). Thus, RAB7 was present in EVs and it was about 3 times more abundant in CDDP resistant cells, in parallel to the increased amount of EVs. To investigate the reasons for RAB7 downregulation in chemoresistant cells, we analyzed the level of RAB7 mRNA by qPCR in A2780 and A2780 CIS cells, but we did not find any difference between the two cell lines (Figure S3a). Thus, we investigated if RAB7 downregulation in A2780 CIS cells was due to an increase of proteasomal degradation or to increased stability of this small GTPase in CDDP sensitive A2780 cells, by inhibiting the proteasome with MG132 or protein translation with cycloheximide, respectively. Again, we found no differences between chemoresistant and chemosensitive A2780 cells (figure S3b-d). Finally, we treated A2780 CIS cells with GW4869 to inhibit EV secretion. As expected, we observed a significant increase of CD81 but also of RAB7 intracellular amount (Fig. 5I, J), confirming that the intracellular quantity of RAB7 was regulated by EV secretion. An increase of RAB7 in EVs was confirmed also by western blot analysis on immunoprecipitated EVs (Fig. 5K, L). In particular, the analysis confirmed a decrease of RAB7 expression in chemoresistant cells accompanied by an increase of RAB7 amount in EVs derived from these cells (Fig. 5L). Altogether these results explain why the amount of RAB7 was significantly decreased in A2780 CIS cells compared to CDDP-sensitive cells, and demonstrate the existence of a new mechanism of regulation of the intracellular levels of RAB7, through the secretion with the EVs.

### CDDP-resistant A2780 CIS cells secrete MDVs as a consequence of late endocytic compartment dysfunction

We observed impairment of mitochondrial function in CDDP-resistant cells, but also impairment of the late endocytic pathway with extrusion of RAB7 by EV secretion. Thus, we hypothesized that MDVs would be secreted from resistant cells rather than delivered to lysosomes for degradation [4, 5]. We analyzed EVs for mitochondrial cargoes and established that CD63, CD9, and CD81 positive EVs were characterized by an increase of mitochondrial ATP synthase subunit alpha (ATP5A), succinate dehydrogenase subunit B (SDHB), and SDHA proteins in chemoresistant cells compared to control A2780 cells. The increase of these proteins amount was found not only in EVs but also in cells, although more limited (Fig. 5K, L-P). In contrast, a significant reduction of NADH:Ubiquinone Oxidoreductase Core Subunit S3 (NDUFS3) expression was found in cell lysates of A2780 CIS cells coherently with the observed mitochondrial bioenergetic defects (Fig. 5K and N). Interestingly, an increased amount of NDUFS3 was instead detected in EVs from chemoresistant cells (Fig. 5K and N). Finally, we extracted total DNA from EVs and mitochondrial DNA (mtDNA) amplification was performed to analyze the occurrence of mtDNA into EVs. As shown in Fig. 5Q, mtDNA was present in EVs derived from both cell lines, but much more abundantly in EVs from A2780 CIS cells.

**Fig. 6:**
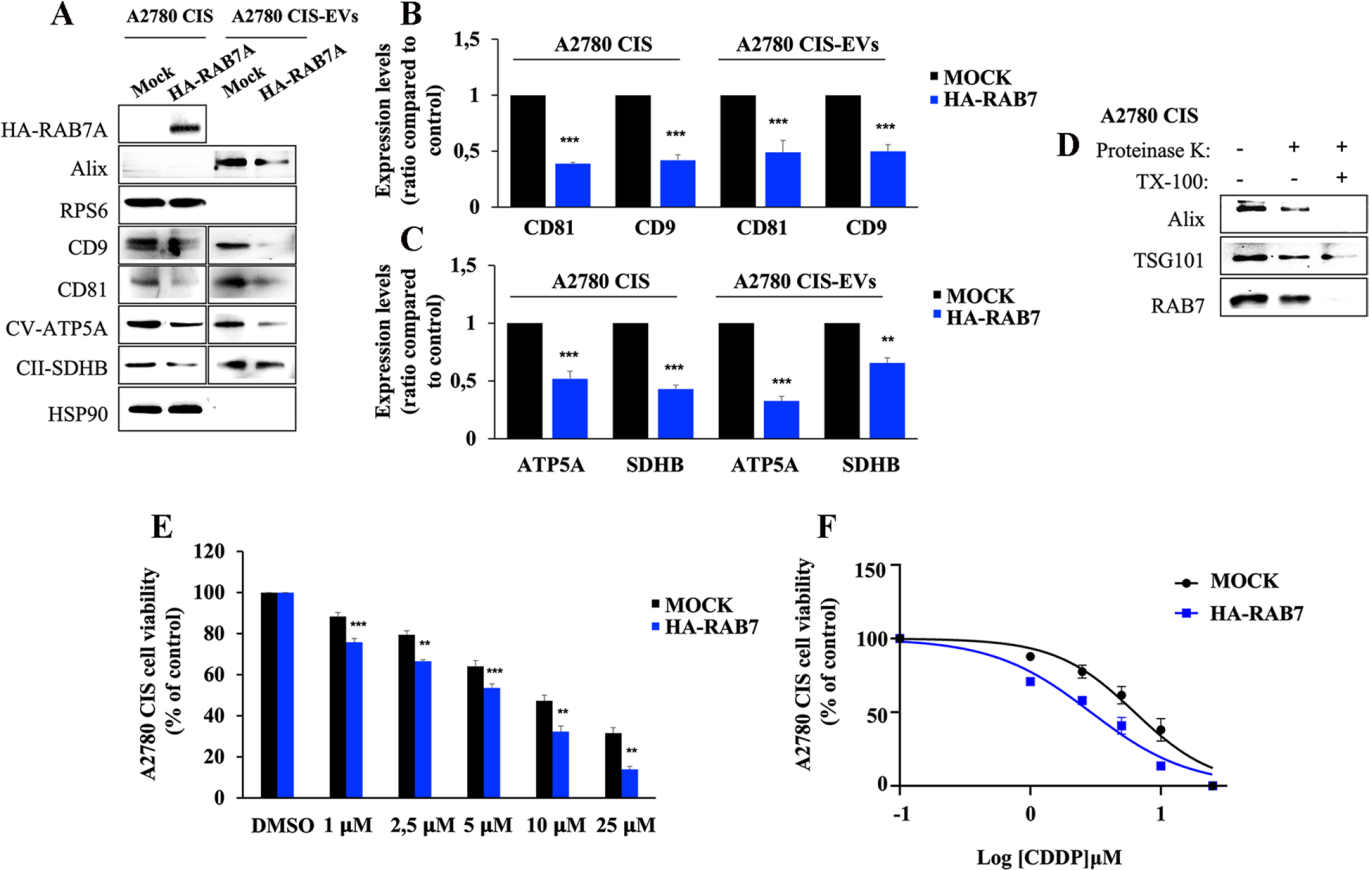
Analysis of EVs after RAB7 overexpression in A2780 CIS cells. **A** A2780 CIS cells were transfected with empty vector (Mock) and with HA-RAB7, and EVs were purified by immunoisolation. Expression levels of CD9, CD81, ATP5A, and SDHB were evaluated by Western blot in transfected cells, and in EVs purified from these cells. **B**, **C** Protein relative abundance was determined through densitometric analysis normalizing against HSP90 protein. **D** EVs from A2780 CIS were incubated with proteinase K where indicated in the presence or absence of Triton-X100 and then were subjected to immunoblot analysis using antibodies against Alix, TSG101 (as controls), and RAB7. **E** A2780 CIS cells transfected with empty vector (Mock) and with HA-RAB7 plasmid were treated with CDDP. Subsequently, an MTT assay was performed and cell viability of HA-RAB7-transfected A2780 CIS cells was determined using the matched mock for each concentration as control. **F** EC50 was determined in A2780 CIS cells treated as indicated after 48 h of incubation with CDDP using the MTT assay and calculated with GraphPad Prism. Data represent the mean ± SEM of at least three independent experiments. * *p* < 0.05; ** *p* < 0.01; ***p < 0.001

In order to establish if late endocytic defects caused by decreased cellular amounts of RAB7 affect MDVs, we overexpressed HA-tagged RAB7 in A2780 CIS cells and looked at EVs and at the presence of mitochondrial proteins in cells and EVs. We found a decrease in the amount of CD9 and CD81 tetraspanins in EVs suggesting a reduced production of EVs (Fig. 6A, B). Moreover, we observed a decrease of ATP5A and SDHB proteins in cells and in EVs purified from HA-RAB7 transfected cells compared to the control (Fig. 6A-C).

To check whether RAB7 is a cargo or trafficking guide of MDVs, we performed a proteinase K protection assay on A2780 CIS cells [37] (Fig. 6D). This assay is based on the concept that proteinase treatment of EVs will cleave RAB7 localized on limiting EV membrane, while RAB7 present in the lumen will be resistant to protease treatment. EVs were treated with proteinase K in the absence or presence of Triton-X100 which solubilizes the EV membranes and is a positive control for the RAB7 total cleavage. Moreover, we used Alix and Tumor Susceptibility Gene 101 (TSG101) as cargo controls [35]. The results showed that RAB7 is present mainly inside EVs as Alix and TSG101 (Fig. 6D). Finally, in order to confirm the pivotal role of RAB7 in the chemoresistant phenotype, we treated A2780 CIS cells with step-wise concentrations of CDDP after RAB7 overexpression (Fig. 6E). We observed a significant increase of CDDP sensitivity at all concentrations compared to matched mock cells. Thus, we demonstrated that overexpression of small GTPase determines a significant decrease in EC50 (Fig. 6F; Table 1).

**Table 1:** Cisplatin (CDDP) EC50 values in A2780 and A2780 CIS cell lines, in A2780 CIS transfected with 2xHA-RAB7A and 2xHA-pcDNA3.1 vector, in A2780 cells after treatment with conditioned media and A2780 CIS EVs and with A2780 EVs, and in SKOV-3, SKOV-3 CIS-1 and CIS-2 resistant clones

	EC50 (µM)
A2780	0,9875 ± 0,11 µM
A2780 CIS	7,2297 ± 0,7 µM (**)
A2780 CIS 2xHA-pcDNA3.1	7,92 ± 0,47 μm
A2780 CIS 2xHA-RAB7A	3,25 ± 0,48 μm (**)
A2780 CIS CM	5,5397 ± 0,21 µM (***)
A2780 CIS EVs	5,6153 ± 0,48 µM (***)
A2780 CIS CM W/O EVs	1,3923 ± 0,16 µM (*)
A2780 CIS GW4869 CM	1,1575 ± 0,08 µM
A2780 EVs	0,894 ± 0,11 µM
A2780 CCCP EVs CM	1,15 ± 0,12 µM (*)
SKOV-3	2,11 ± 0,27 µM
SKOV-3 CIS-1	12,01 ± 0,75 µM (***)
SKOV-3 CIS-2	8,62 ± 0,13 µM (***)

*Abbreviations*: *EC50* half-maximal effective concentration, *CM* Conditioned Medium, *CCCP* cyanide m-chlorophenylhydrazone

* *p* < 0.05, **p < 0.01, ***p < 0.001

Altogether, these results demonstrate that EVs from chemosensitive and chemoresistant cells contain mitochondrial cargoes, and these mitochondrial cargoes, both in terms of protein and mtDNA, are more abundant in EVs from chemoresistant cells. The increased secretion of MDVs by chemoresistant cells is ultimately caused by the intracellular decrease of RAB7, which becomes the driver cargo of MDVs, and impairs the late endocytic pathway.

### Mitochondrial bioenergetic deficit may induce extrusion of RAB7 by EV secretion

**Fig. 7:**
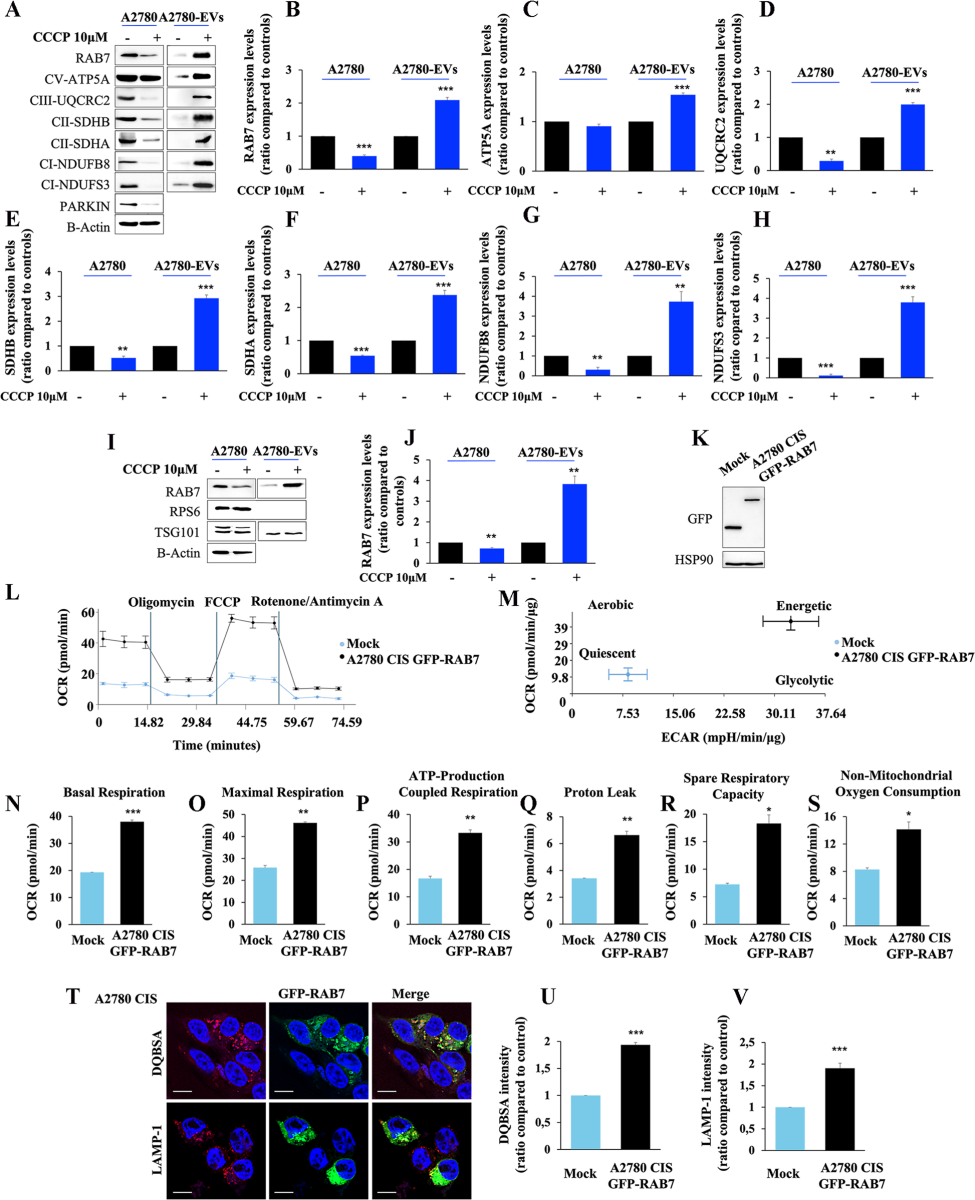
Treatment with carbonyl cyanide m-chlorophenylhydrazone (CCCP) and analysis of EV content. **A** A2780 cells were treated with CCCP (10 μm) and EVs were purified by immunoisolation. Mitochondrial protein expression was evaluated in EVs by Western blot analysis and **B**-**H** the relative abundance of RAB7, ATP5A, UQCRC2, SDHA, SDHB, NDUFS3, and NDUFB8 was determined through densitometric analysis normalizing against β-Actin. Parkin protein was used as control of CCCP treatment. **I**,** J** A2780 cells were treated with CCCP (10 μm) and EVs were purified by ultracentrifugation. RAB7 levels were determined, and their relative abundance was measured with densitometry normalizing against β-Actin. **K** A2780 CIS cells were transfected with empty vector (Mock) and with GFP-RAB7, and **L** OCR was determined with Seahorse Mito stress kit assay. **M** OCR and Extracellular Acidification Rate (ECAR) obtained with Seahorse instruments allowed to determine the energetic map. **N**-**S** Basal respiration, maximal respiration, ATP-production coupled respiration, proton leak, spare respiration capacity, and non-mitochondrial oxygen consumption were determined by Seahorse data elaboration. **T**-**V** A2780 CIS cells were transfected with GFP-RAB7, and DQBSA assay and LAMP-1 immunostaining were performed and analyzed by confocal microscopy. Data represent the mean ± SEM of at least three independent experiments. * *p* < 0.05; ** *p* < 0.01; ***p < 0.001

In order to understand the cause-effect relationship among mitochondrial and lysosomal dysfunctions, increased amounts of RAB7 within EVs, and increased MDV secretion in CDDP-resistant cells, we treated A2780 cells with carbonyl cyanide *m*-chlorophenyl hydrazone (CCCP). CCCP is a well-known mitochondrial oxidative phosphorylation uncoupler, able to induce mitochondrial dysfunction. In this experiment, we used CDDP-sensitive A2780 cells because both the late endocytic pathway and mitochondria are functional in these cells, and this is a necessary prerequisite to analyze the effects of CCCP on mitochondria, lysosomes, EVs, and MDVs. Interestingly, we observed a significant reduction of cellular RAB7 and a significant increase of RAB7 in EVs upon CCCP treatment (Fig. 7A, B) indicating that the mitochondrial bioenergetic deficit triggers RAB7 secretion through EVs. Moreover, to see if MDV secretion is also induced by CCCP-treated dysfunctional mitochondria, we analyzed the mitochondrial signature of EVs upon CCCP-treatment, and we found an increase of several mitochondrial proteins, such as ATP5A, Ubiquinol-Cytochrome C Reductase Core Protein 2 (UQCRC2), SDHB, SDHA, NADH:Ubiquinone Oxidoreductase Subunit B8 (NDUFB8) and NDUFS3 (Fig. 7A, C-H). Experiments performed on EVs purified by ultracentrifugation confirmed the increase of RAB7 inside EVs after CCCP treatment (Fig. 7I, J). Altogether, these results indicate that mitochondrial bioenergetic deficit may induce lysosomal dysfunction by decreasing RAB7 cellular amount and suggest that the mitochondria-lysosome crosstalk modulates EV and MDV secretion in response to CDDP treatment. Moreover, we induced rescue of RAB7 expression in A2780 CIS cells through GFP-RAB7 wild-type transfection and we measured mitochondrial metabolism by Seahorse analysis (Fig. 7K, L-S). Coherently with our hypothesis, we observed an increase in mitochondrial activity in A2780 CIS GFP-RAB7 transfected cells compared to mock cells (Fig. 7L) with a shift from quiescent to energetic metabolism (Fig. 7M). Indeed, OCR was increased with all analyzed parameters, such as basal and maximal respiration, ATP production, proton leak, spare respiratory capacity, and non-mitochondrial OCR (Fig. 7N-S). Thus, RAB7 is able to regulate mitochondrial function compensating MQC alteration, and the secretion of this small GTPase embedded in EVs has a pivotal role in MQC deficit.

Finally, we analyzed lysosomal activity and abundance in A2780 CIS cells using DQBSA assay and LAMP-1 immunostaining upon GFP-RAB7 wt expression (Fig. 7T-V). As expected, we observed increased fluorescent intensity of both evaluated signals in transfected compared to mock cells, indicating that RAB7 is essential to lysosomal functions in CDDP-resistant cells.

#### EVs secreted by CDDP-resistant A2780 CIS cells confer phenotypic resistance to CDDP-sensitive A2780 cells

**Fig. 8:**
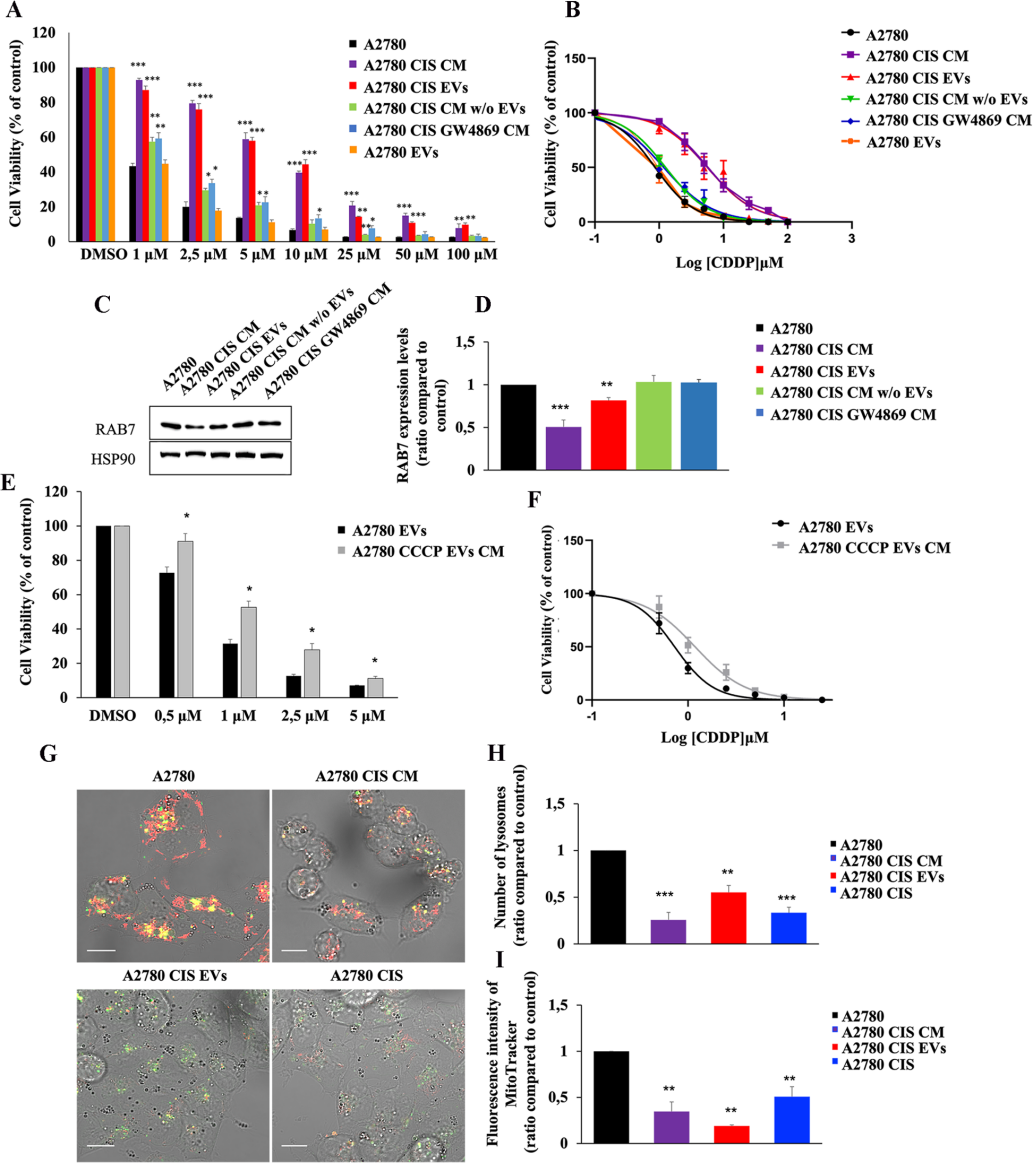
Mitochondria-Derived Vesicles influence chemotherapy response. **A** A2780 cells were incubated with media or conditioned media (CM) as indicated (A2780 CIS CM, A2780 CIS EVs, A2780 CIS CM w/o EVs, A2780 CIS GW4869 CM, and A2780 EVs), and were treated with stepwise concentrations of CDDP. Then, cell viability was measured with the MTT assay. **B** EC50 was determined in A2780 cells treated as indicated after 72 h of incubation with CDDP using the MTT assay and calculated with GraphPad Prism. **C** Lysates from A2780 cells incubated with media or conditioned media (CM) as indicated (A2780 CIS CM, A2780 CIS EVs, A2780 CIS CM w/o EVs, and A2780 CIS GW4869 CM) were analyzed by immunoblotting using antibodies against RAB7 and HSP90, as the loading control. **D** Densitometric analysis was performed by Image Lab software (Bio-Rad). **E** A2780 cells were incubated with media or conditioned media (CM) as indicated (A2780 EVs and A2780 CCCP EVs CM) and were treated with stepwise concentrations of CDDP. Then cell viability was measured with the MTT assay. **F** EC50 was determined in A2780 cells treated as indicated after 72 h of incubation with CDDP using the MTT assay and calculated with GraphPad Prism. **G** Live imaging assay using LysoTracker DND-26 and MitoTracker Red CM-H_2_XRos on A2780 cells incubated with A2780 CIS CM and A2780 CIS EVs and on A2780 CIS cells used as control. **H**-**I** Number of lysosomes for cells and intensity of MitoTracker Red CM-H_2_XRos were determined by ImageJ software. Data represent the mean ± SEM of at least three independent experiments. * *p* < 0.05; ** *p* < 0.01; ***p < 0.001. Scale bar = 10 μm

The nature of CDDP resistance is multifactorial. In addition to genetic mechanisms also epigenetic mechanisms are involved [13–18], and, among the latter, interactions between CDDP-sensitive and CDDP-resistant cells in the tumor microenvironment may play a relevant clinical role. In this context, we investigated the possible involvement of EV secretion in the mechanism of chemoresistance induction. We treated A2780 chemosensitive cells with different conditioned media (CM) or with purified EVs. In particular, we used the following media: (i) A2780 CIS CM, collected from A2780 CIS cells (ii) A2780 CIS EVs, obtained by adding EVs purified from the CM of A2780 CIS cells to fresh medium (iii) A2780 CIS w/o EVs, obtained by removing, from the CM of the A2780 CIS cells, EVs by ultracentrifugation; (iv) A2780 CIS GW4869, obtained after treatment of A2780 CIS cells with GW4869, an inhibitor of EV secretion and, finally, (v) A2780 CM, obtained from A2780 cell lines, used as further negative control. A2780 cells were incubated for 96 h with these different media and treated with increasing stepwise concentrations of CDDP. Interestingly, we observed a significant increase in cell viability from 1µM to 25 µM of CDDP only in A2780 cells grown in media containing EVs derived from A2780 CIS cells (Fig. 8A). Indeed, similar results were obtained by incubating CDDP sensitive cells with conditioned medium derived from A2780 CIS cells or with a fresh medium where EVs purified from A2780 CIS cells were added. In contrast, sensitivity to CDDP did not change in A2780 cells grown in conditioned medium from A2780 CIS cells deprived of EVs, in the conditioned medium of A2780 CIS cells treated with GW4869 and in A2780 cells grown in media containing EVs derived from A2780 cells (Fig. 8A) demonstrating that EVs were responsible for the acquisition of chemoresistance. Half-maximal effective concentration (EC50) value is used as a measure of the drug’s potency. We have analyzed EC50 in A2780 cells treated with A2780 CIS conditioned medium or with EVs purified from A2780 CIS cells. We found that EC50 values in A2780 cells treated with A2780 CIS CM or with EVs purified from A2780 CIS cells were significantly higher compared to A2780 cells, being comparable to the EC50 of A2780 CIS cells (Table 1; Fig. 8B). In contrast, EC50 values of A2780 cells treated with conditioned medium of A2780 CIS cells deprived of EVs, incubated with GW4869, or incubated with A2780 EVs were similar to that obtained for control A2780 cells (Table 1; Fig. 8B).

To understand whether the acquisition of the chemoresistant phenotype in A2780 cells treated EVs from A2780 CIS cells was accomplished by RAB7 downregulation, we analyzed expression levels of this small GTPase after treatment with (i) conditioned medium from A2780 CIS cells, (ii) EVs derived from A2780 CIS cells, (iii) conditioned medium from A2780 CIS cells deprived of EVs and conditioned medium derived from A2780 CIS cells treated with GW4869 (Fig. 8C, D). Consistent with previous experiments, we observed a significant decrease of RAB7 expression in A2780 cells treated with A2780 CIS CM or treated with EVs from chemoresistant cells.

In order to assess the role of MDVs in the acquisition of chemoresistance, we checked whether MDVs derived from CCCP-treated A2780 cells could induce a chemoresistant phenotype in A2780 chemosensitive cells (Fig. 8E). Interestingly, we found that treatment of these cells with CM derived from CCCP-treated A2780 cells induced chemoresistant behavior compared to control cells. A significant increase in EC50 confirmed this result (Fig. 8F; Table 1).

To further investigate the effects caused by treatment with EVs from A2780 CIS cells, we performed live-cell imaging using LysoTracker DND-26 and MitoTracker Red CM-H_2_XRos in A2780 cells incubated with CM from A2780 CIS cells (A2780 CIS CM) and with medium containing EVs purified from A2780 CIS cells (A2780 CIS EVs) (Fig. 8G). A2780 CIS cells were used as control (Fig. 8G). Analysis of lysosomal abundance revealed that treated A2780 cells undergo a decrease in the number of lysosomes becoming comparable to A2780 CIS resistant cells (Fig. 8H). Finally, we also observed a reduction in mitochondrial activity (Fig. 8I). Indeed, the staining with a MitoTracker probe, characterized by the ability to fluoresce upon oxidation and by the fact that its accumulation is dependent on membrane potential, was lower in A2780 cells treated with EVs or conditioned medium from A2780 CIS cells and, notably, quantification revealed even lower values compared to A2780 CIS cells.

**Fig. 9:**
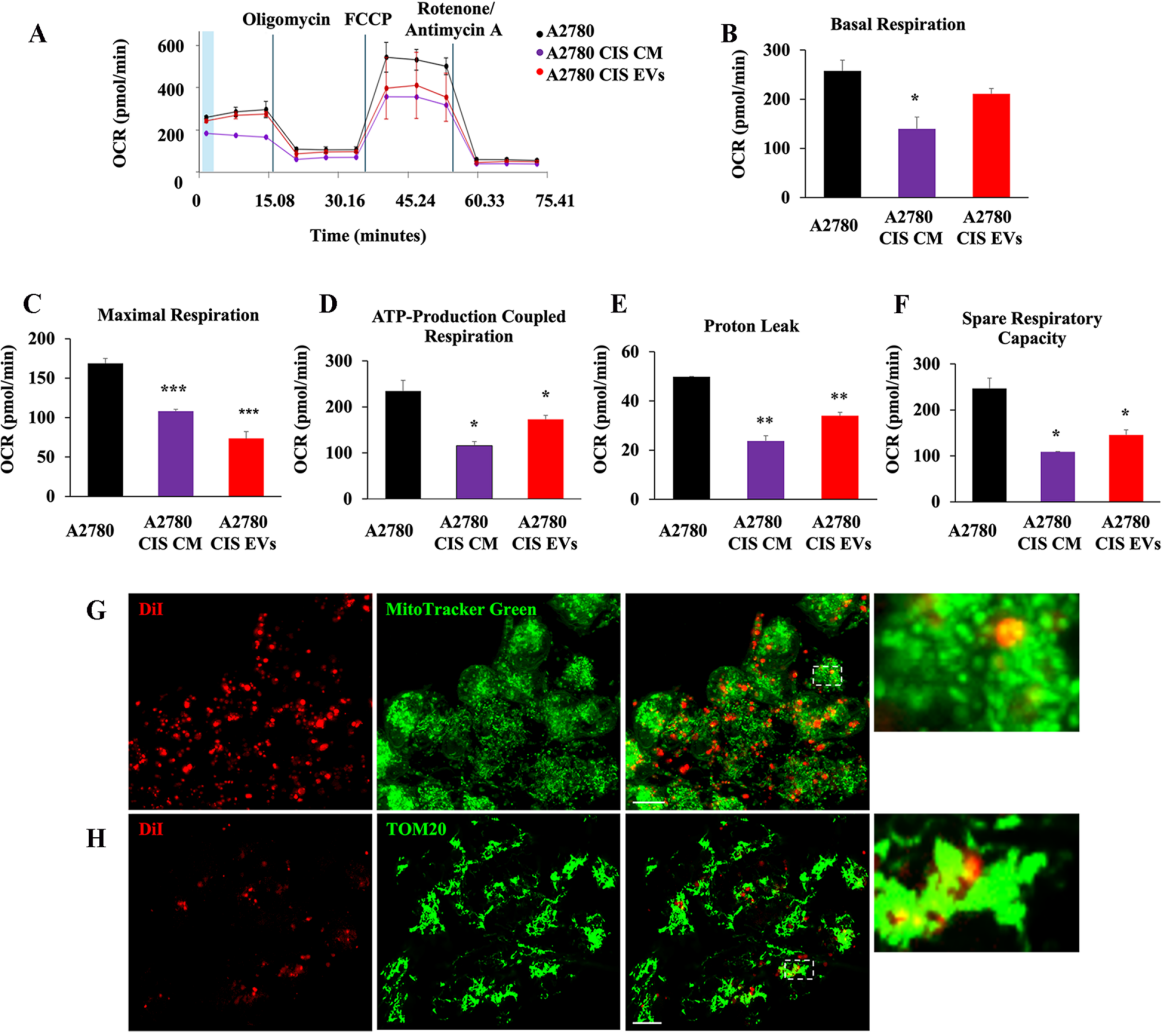
Mitochondria-derived vesicles induce mitochondrial bioenergetic deficit in recipient chemosensitive cells. **A** Oxygen rate consumption (OCR) was determined in A2780 and A2780 treated with A2780 CIS CM and A2780 CIS EVs with Seahorse Mito stress kit assay. **B**-**F** Basal respiration, maximal respiration, ATP-production coupled respiration, proton leak, and spare respiration capacity were determined by Seahorse data elaboration. **G**-**H** DiI-stained EVs were detected co-localizing with mitochondria in A2780 recipient cells by live-cell imaging assay using MitoTracker Green and immunofluorescence using anti-TOM20 antibody. Data represent the mean ± SEM of at least three independent experiments. * *p* < 0.05; ** *p* < 0.01; ***p < 0.001. Scale bar = 10 μm

In light of this, we have performed the Seahorse assay in A2780 cells treated with A2780 CIS CM or with A2780 CIS EVs (Fig. 9A). As we supposed, treatment determines a bioenergetic deficit in A2780 CDDP-sensitive cell line (Fig. 9A-F). Moreover, EV staining with the DiI dye revealed that these vesicles are directed to A2780 mitochondria, and we observed quasi-total co-localization between DiI-stained EVs and mitochondria of recipient cells with both live imaging assay (Fig. 9G) and immunofluorescence (Fig. 9H). Altogether these results demonstrate that EVs secreted by A2780 CIS are able to induce changes in mitochondria-lysosome crosstalk and, more importantly, CDDP resistance in otherwise sensitive cells.

### MDVs were secreted as a result of defects of mitochondria-lysosomes communication also in SKOV3 CDDP-resistant ovarian cancer cells

**Fig. 10:**
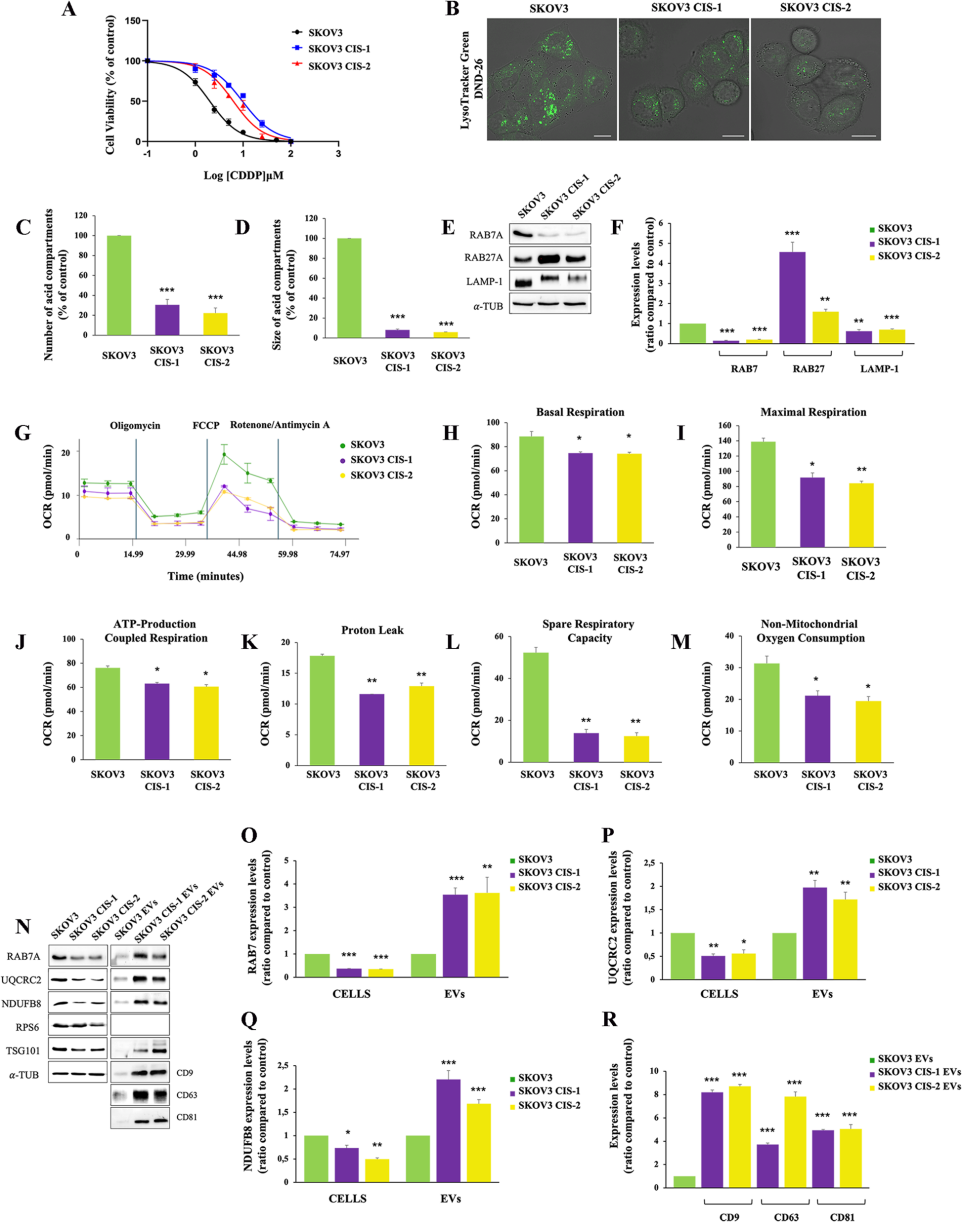
Analysis of mitochondrial and lysosomal deficit and EVs in SKOV-3 sensitive and resistant cells. **A** EC50 was determined in SKOV-3, SKOV-3 CIS-1, and SKOV-3 CIS-2 after 72 h of treatment with CDDP using the MTT(3-(4,5-dimethylthiazol-2-yl)-2,5-diphenol tetrazolium bromide) assay. **B** Late endocytic acid compartments were live stained with LysoTracker DND-26 dye and images were acquired with confocal microscopy. **C**,** D** The number and size of LysoTracker-positive organelles were determined by ImageJ software. **E**,** F** Relative protein abundance of RAB7, RAB27, and LAMP-1 was assessed by Western blot analysis and quantified by densitometry normalizing against α-Tubulin. **G** Oxygen rate consumption (OCR) was determined in SKOV-3 and in SKOV-3 CIS-1 and CI-2 with Seahorse Mito stress kit assay. **H**-**M** Basal respiration, maximal respiration, ATP-production coupled respiration, proton leak, and spare respiration capacity were determined by Seahorse data elaboration. **N**-**R** EVs were purified by immunoisolation. Positive and negative controls were used to verify the purity of EVs. Mitochondrial protein expression was evaluated in EVs by Western blot and the relative abundance of RAB7, UQCRC2, NDUFB8, and three tetraspanins (CD9, CD63, and CD81) was determined through densitometric analysis normalizing against α-Tubulin.* *p* < 0.05; ** *p* < 0.01; ***p < 0.001. Scale bar = 10 μm

In order to confirm our data in another cellular model, we induced chemoresistance in SKOV3 ovarian cancer cell line with prolonged exposure to CDDP [23], obtaining two resistant clones, named SKOV3 CIS-1 and SKOV3 CIS-2 (Table 1; Fig. 10A). In these cells, we performed live cell imaging using Lysotracker dye (Fig. 10B) and we found a significant reduction in lysosomal number and size (Fig. 10C, D) as observed in A2780 CIS cells. We also observed a significant decrease of RAB7 expression in both clones accompanied by LAMP-1 downregulation and an increase in RAB27A expression (Fig. 10E, F). Mitochondrial bioenergetic deficit occurred also in SKOV3 resistant cells as shown in Fig. 10 (G-M) demonstrating the occurrence of defects in lysosomes and mitochondria also in this case. Then, we purified EVs by immunoprecipitation from SKOV3 and the resistant counterparts and we observed, coherently with RAB27A upregulation, increased secretion of EVs with RAB7 and mitochondrial cargoes (Fig. 10N-R).

Altogether these results confirm our proposed model of CDDP chemoresistance based on RAB7 intracellular downregulation, mitochondrial/lysosomal dysfunction, and EV secretion as markers and propagators of chemoresistance.

## Discussion

In this study, we discovered a novel mechanism of resistance to cisplatin (CDDP), in which MDV secretion plays a central role. CDDP can enter into the cells through different routes and several mechanisms of chemoresistance may occur. The formation of DNA adducts upon CDDP binding to the nucleophilic N7 sites of purines is the well-characterized model of CDDP action through which this drug is able to induce DNA damage response and apoptosis [11, 12]. General mechanisms of chemoresistance are due to reduced DNA damage, as a consequence of an increase in DNA adduct repair, reduced drug uptake, or increased drug inactivation [13–16].

Nevertheless, multiple factors, such as genetic changes, epigenetic alterations at both molecular and cellular levels, and heterogeneity among cancer cells, can influence the activation of these mechanisms [15, 17, 18]. For instance, it was observed that CDDP can accumulate into lysosomes to induce cell death through lysosomal membrane permeabilization (LMP) [72]. In contrast, lysosomes can also sequester CDDP avoiding chemotherapeutic drugs to reach their targets and determining chemoresistance occurrence [73]. Furthermore, mitochondrial DNA (mtDNA) is primarily targeted by CDDP to induce apoptosis, to the detriment of CDDP binding to nuclear DNA, which becomes limited [74–76]. Interestingly, mtDNA alterations have been implicated in the development of cancer and chemoresistance [74, 75, 77–80]. Indeed, we have observed, in a tumor-resistant mass from a patient treated with CDDP-derived compound, a shift to homoplasmy of a mtDNA mutation in a mitochondrial complex I gene and accumulation of dysfunctional mitochondria [41]. Moreover, it has been demonstrated that mtDNA mutations and alterations in mitochondrial functions characterize ovarian cancer cells resistant to CDDP [81]. Finally, it may be also noted that the CDDP-resistant A2780 CIS cell line, which we used as a model in this study, harbors the K351N substitution in p53 that has been shown to impair ubiquitination of p53, and CDDP-induced translocation of p53 to mitochondria [49], while SKOV-3 are described as p53-null cell lines [82].

These premises led us to hypothesize the changes in mitochondria-lysosome crosstalk leading to dysfunctional MQC might be involved in acquired CDDP-resistance in ovarian cancer cells. MQC is central in cell homeostasis, and several MPQ pathways have been identified which can be classified into: (i) mitochondrial morphology control (fission and fusion), (ii) mitophagy, (iii) micromitophagy and (iv) mitochondrial protease system [83]. In the process of mitophagy, dysfunctional mitochondria are first detected, then separated from the mitochondrial network, and recruited by the mitophagosome. In contrast, during micromitophagy damaged mitochondria can generate MDVs that are internalized into the lysosomal lumen for degradation. An alternative micromitophagy mechanisms, which is known as mitochondria-eating protein (Mieap)-induced accumulation of lysosome-like organelles within mitochondria (MALM), is characterized by the transfer of lysosomal proteins into mitochondria to destroy the oxidized mitochondrial protein. When mitochondria are severely damaged or MALM is inhibited, Mieap-induced vacuoles (MIVs) uptake the entire damaged mitochondria into the lysosome for decay. In all of these pathways RAB7 plays a key role by interacting with damaged mitochondria through contact sites [84]. Therefore, changes in RAB7 regulation result in changes in MQC processes.

We previously demonstrated that RAB7 downregulation, and consequent late endocytic impairment, occurred in several CDDP resistant cells with consequent CDDP efflux due to an increase in extracellular vesicle (EV) secretion. Moreover, we demonstrated that modulation of RAB7 expression was able to determine different chemoresistant behavior and, contextually, RAB7 regulated the secretion of EVs that was increased in chemoresistant cells [23]. In this work, we found that ovarian cancer cells resistant to CDDP, compared to their sensitive counterparts, are also characterized by defective lysosomes and mitochondria and we have now more deeply investigated the mechanism of chemoresistance in these cells.

First of all, CDDP resistant ovarian cells were characterized by a lower number and size of acid compartments, by the lower expression of a number of endocytic and lysosomal proteins, and by a bioenergetic mitochondrial deficit as measured by decreased mitochondrial respiration and ATP production. Thus, we confirmed and extended previous findings obtained on different cell lines as mitochondrial and lysosomal defects have been already demonstrated to be a feature of CDDP resistant cancer cells [23, 41, 79, 85, 86].

Second, we observed for the first time that EV secretion represents a novel mechanism to control intracellular RAB7 amount. Indeed, although we observed a consistent decrease of RAB7 expression in chemoresistant cells, this downregulation does not seem to be due to transcriptional or translational changes and/or to alteration of protein degradation, as no differences were found between chemoresistant and chemosensitive cell lines in the mRNA or upon treatment with MG132 or with cycloheximide. Instead, the observed decrease of RAB7 inside chemoresistant cells was accompanied by an increase in the abundance of RAB7 cargo in EVs actually paralleling the increase of EV secretion in chemoresistant cells. The presence of RAB proteins in EVs has previously sporadically reported although no explanation about why RABs should be included in EVs has been given [87–89]. We suppose that the endocytic dysfunction caused by RAB7 downregulation offers an adaptative and unique route toward endosomal vesicle secretion to chemoresistant cells.

Third, the investigation of EV content revealed that a mitochondrial signature characterizes EVs of chemoresistant cells both in terms of proteins and mtDNA. We demonstrated that impairment of the late endocytic pathway induced by downregulation of RAB7 in chemoresistant cells determines, in turn, a deviation of the degradative route of MDVs which are secreted in extracellular space in an attempt to ensure the MQC. Thus, MDVs are secreted instead of being targeted for degradation in lysosomes. Furthermore, the increase of RAB9 and RAB27 expression, the decrease of Parkin expression, and the impairment of the mitophagy pathway corroborated our hypothesis. It is not negligible that CDDP can reach multiple targets inside the cells and in the ovarian cancer context it is able to induce synergistic damage to acid organelles and mitochondria. In our opinion, the chemoresistant phenotype is due not only to the ability to increase the efflux of CDDP through EVs but also to the acquisition of a selective advantage by the cells becoming capable of eliminating mitochondrial damaged particles through MDV secretion.

Indeed, in CDDP-resistant A2780 CIS cells the mitochondrial and lysosomal turnover through the mTOR-TFEB axis, notoriously implied in lysosomal/mitochondrial biogenesis and in autophagy and mitophagy pathways [66–70], is significantly compromised, possibly as a consequence of the p53 mutation that may affect the complex cross-talk between p53 and mTOR pathway overseeing MQC and lysophagy [90, 91]. Interestingly, LMP is essential for induction of apoptosis and p53 regulates LMP [91]. This may result in increased mitochondrial senescence and MDV secretion. Our hypothesis is confirmed by the occurrence of similar effects observed when we treated sensitive cells with an inhibitor of mitochondrial function, such as the CCCP. Indeed, treatment with CCCP causes mitochondrial impairment, RAB7 downregulation, and an increase in MDVs. Moreover, chemosensitive cells acquire chemoresistant phenotype after treatment with MDV derived from A2780 CCCP-treated cells.

Fourth, we have demonstrated that RAB7-signed MDVs coming from chemoresistant cells are able to induce chemoresistance. Indeed, notably, when sensitive ovarian cancer cells were grown in media containing RAB7-signed MDVs from chemoresistant cells, a switch toward the chemoresistant phenotype was observed. In addition, these vesicles were also able to induce defective mitochondrial phenotype in recipient sensitive cell lines. Although the molecular mechanisms underlying this phenomenon remain unclear, these data prove the importance of MDVs and in particular the importance of tumor microenvironment in the acquisition of chemoresistance.

## Conclusions

**Fig. 11:**
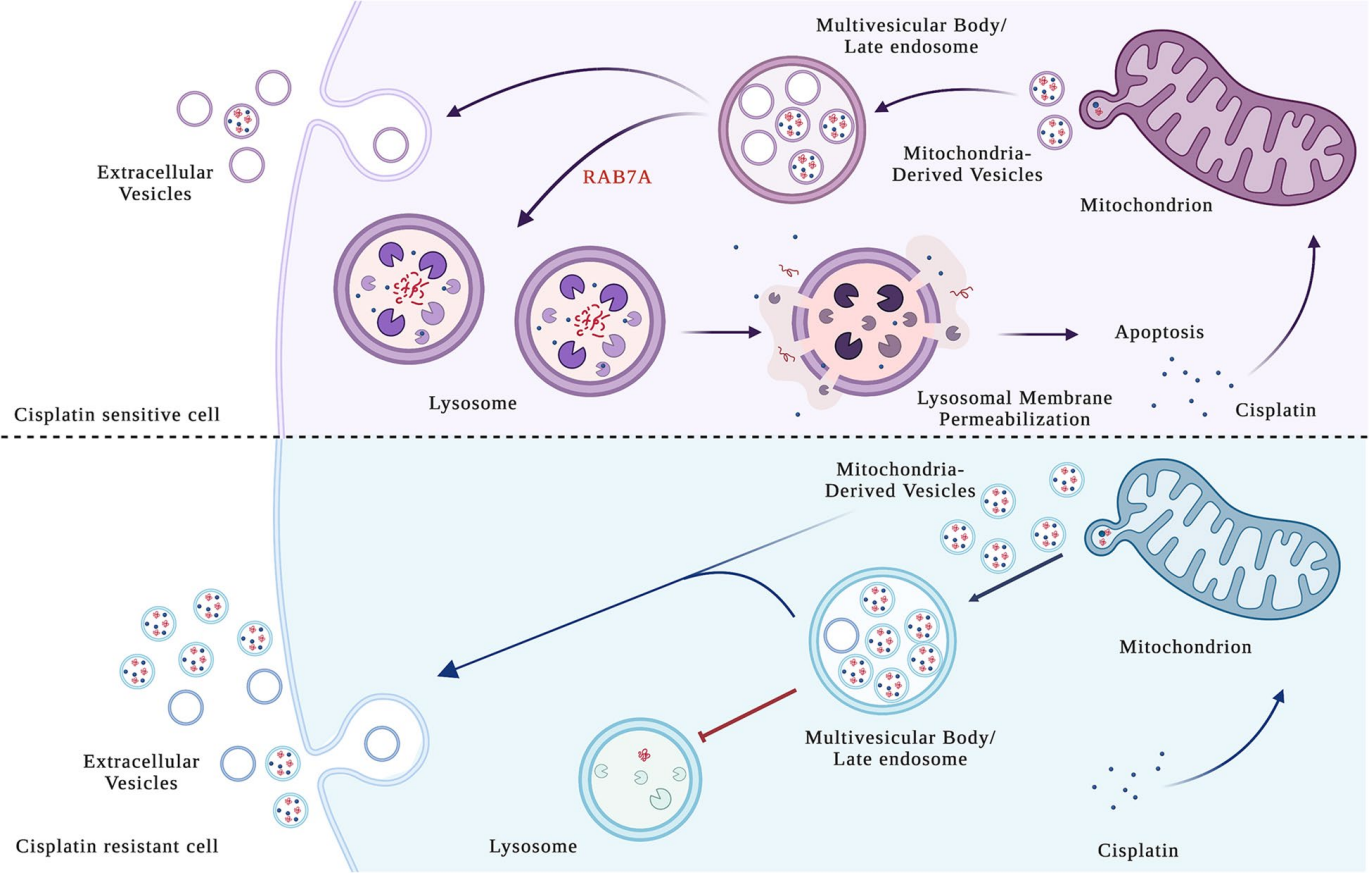
Proposed mechanism of chemoresistance MDV-mediated. In chemosensitive cells, cisplatin uptake induces mitochondrial damage and generation of MDVs which are destined to MVBs and then degraded in lysosomes, or, alternatively, to a limited extent secreted. Under these conditions, cisplatin determines lysosomal membrane permeabilization (LMP) and cell death by apoptosis. In chemoresistant cells, mitochondria are dysfunctional and the cell responds to cisplatin increasing the number of MDVs. This cell is also characterized by a lysosomal deficit, and this condition determines massive secretion of MDVs outside the cells to propagate chemoresistance in the cancer microenvironment

 We conclude that CDDP-resistance, in the specific cellular context of ovarian cancer, can be due to concomitant deficit of lysosomal and mitochondrial functions compromising their essential communications. In resistant cells in which the MQC is compromised, a compensatory mechanism determines an increase of MDVs secretion whose propagation in the tumor microenvironment could become one of the cause of chemoresistance (Fig. 11).

In this study, we demonstrated that this synergistic deficit is due to the downregulation of RAB7, a protein that is essential for lysosomal function, biogenesis, acidification, and localization [19], but also for ancillary functions, such as mitophagy, to eliminate damaged mitochondria [27]. We have observed that secretion of RAB7-signed MDVs is a new regulation mechanism of RAB7. Indeed, as shown in Fig. 11, in CDDP-sensitive cells, RAB7 is able to guarantee endocytic function, while CDDP uptake in lysosomes determines lysosomal membrane permeabilization and, hence, apoptosis [72].

In CDDP-resistant cells, lysosomal deficit prevents mitophagy to eliminate mitochondrial-damaged particles, which are secreted into MDVs as MQC mechanism. Thus, these vesicles vehicle CDDP and mitochondrial damaged particles. The latter, captured by CDDP-sensitive cells, are directed to the mitochondria of sensitive cells and are able to propagate chemoresistance. We hypothesize that mitochondrial deficit is induced by the uptake, inside sensitive cells, of damaged mitochondrial particles triggering mitochondrial deficit, impairment of mitochondria-lysosomes communication, with consequent lysosomal dysfunction, and, thus, chemoresistant phenotype. In addition, signaling molecules and/or non-coding RNA present in these vesicles could alter recipient cell behaviour. In this context, RAB7, as the signature of EVs, could be important for docking and fusion of MDVs with mitochondria [92] of CDDP-sensitive cells, triggering a vicious cycle in which these cells increase secretion of MDVs, being unable to eliminate them with lysosomal degradation.

Our principal perspective will be to study these vesicles in biological fluids from ovarian cancer patients before and after chemotherapy treatment with the aim of better understanding this mechanism of chemoresistance. Moreover, this work provides the basis for hypothesizing a future approach in which platinum compounds are adjuvated by activators of lysosomal and mitochondrial functions to overcome chemoresistance.

### Supplementary Information

**Additional file 1.**

## Data Availability

The data that support the findings of this study are available within the paper and its Supplementary information and/or from the authors upon reasonable request.

## Abbreviations

2-DG: 2-deoxy-D-glucose
ATP: Adenosine Triphosphate
ATP5A: ATP Synthase Subunit Alpha
ATP6V1G1: ATPase H + Transporting V1 Subunit G1
BCA: Bicinchoninic Acid
CDDP: Cisplatin
CHX: Cycloheximide
CLSM: Confocal Laser Scanning Microscope
CM: Conditioned Medium
CMT2B: Charcot-Marie Tooth type 2 B
DCFH-DA: 2′,7′-Dichlorodihydrofluorescein diacetate
DiI: 1,10 -dioctadecyl-3,3,3’,3’-tetramethylindocarbocyanine perchlorate
DLS: Dynamic Light Scattering
DMSO: Dimethyl Sulfoxide
DQBSA: Self-Quenched BODIPY Dye Conjugates of Bovine Serum Albumin
EC50: Half-Maximal Effective Concentration
ECAR: Extracellular Acidification Rate
EV: Extracellular Vesicles
FBS: Fetal Bovine Serum
FCCP: Carbonyl cyanide-p-trifluoromethoxyphenylhydrazone
GAP: GTPase-Activating Protein
GTP: Guanosine Triphosphate
GTPase: Guanosine Triphosphatase
LAMP-1: Lysosomal Associated Membrane Protein 1
LC3B: Microtubule Associated Protein 1, Light Chain 3
LMP: Lysosomal Membrane Permeabilization
MDVs: Mitochondria-Derived Vesicles
MQC: Mitochondrial Quality Control
mtDNA: mitochondrial DNA
mTOR: mammalian Target of Rapamycin
MTT: (3- (4,5-dimethylthiazol-2yl)-diphenyl tetrazolium bromide)
MVBs: Multivesicular Bodies
NDUFB8: NADH:Ubiquinone Oxidoreductase Subunit B8
NDUFS3: NADH:Ubiquinone Oxidoreductase Core Subunit S3
NRF-1: Nuclear Respiratory Factor 1
OCR: Oxygen Consumption Rate
OXPHOS: Oxidative Phosphorylation
PBS: Phosphate Buffer
PCR: Polymerase Chain Reaction
PDI: Polydispersity Index
PGC-1α: Peroxisome-Proliferator-Activated Receptor Coactivator-1α
PINK1: PTEN-Induced Kinase1
PSD: Volume Particle Size Distribution
qRT-PCR: quantitative Real Time PCR
RAB7: Ras-related Brain 7
RILP: Rab-Interacting Lysosomal Protein
RNAi: RNA interference
ROS: Reactive Oxygen Species
RPS6: Ribosomal Protein S6
SDHB: Succinate Dehydrogenase Subunit B
siRNA: small interfering RNA
TBC1D15: TBC Family Member 15
TEM: Transmission Electron Microscopy
TFAM: Mitochondrial Transcription Factor A
TFEB: Transcriptional Factor EB
TSG101: Tumor Susceptibility Gene 101
UQCRC2: Ubiquinol-Cytochrome C Reductase Core Protein 2
