# Supplementary material for: Defects of mitochondria-lysosomes communication induce secretion of mitochondria-derived vesicles and drive chemoresistance in ovarian cancer cells
Source: Cell Commun Signal. 2024 Mar 6;22:165. doi: 10.1186/s12964-024-01507-y (PMC10916030; doi:10.1186/s12964-024-01507-y)
Supplement: Supplementary file 1 — Additional file 1. [file 12964_2024_1507_MOESM1_ESM.docx]

**Defects of mitochondria-lysosomes communication induce secretion of mitochondria-derived vesicles and drive chemoresistance in ovarian cancer cells**

Sinforosa Gagliardi^1^, Marco Mitruccio^1^, Riccardo Di Corato^3,4^, Roberta Romano^1,2^, Alessandra Aloisi^3^, Rosaria Rinaldi^5,6^, Pietro Alifano^1,2^, Flora Guerra^1†^ and Cecilia Bucci^1,2†*^

^1^ Department of Biological and Environmental Sciences and Technologies, University of Salento, Via Provinciale Lecce-Monteroni n. 165, 73100 Lecce, Italy

^2^ Department of Experimental Medicine, University of Salento, Via Provinciale Lecce-Monteroni n. 165, 73100 Lecce, Italy

^3^ Institute for Microelectronics and Microsystems (IMM), CNR, Via Monteroni, 73100, Lecce, Italy

^4^  Center for Biomolecular Nanotechnologies, Istituto Italiano di Tecnologia, Arnesano 73010, Italy

^5^ Department of Mathematics and Physics “E. De Giorgi”, University of Salento, Via Monteroni, Lecce 73100, Italy

^6^ Scuola Superiore ISUFI, University of Salento, Via Monteroni, University Campus, Lecce 73100, Italy

**Correspondence**

*Cecilia Bucci, Department of Experimental Medicine, University of Salento, Via Provinciale Lecce-Monteroni n. 165, 73100 Lecce, Italy

Email: [cecilia.bucci@unisalento.it](mailto:cecilia.bucci@unisalento.it)

^†^ Flora Guerra and Cecilia Bucci are co-last authors.

**Supplementary Materials**


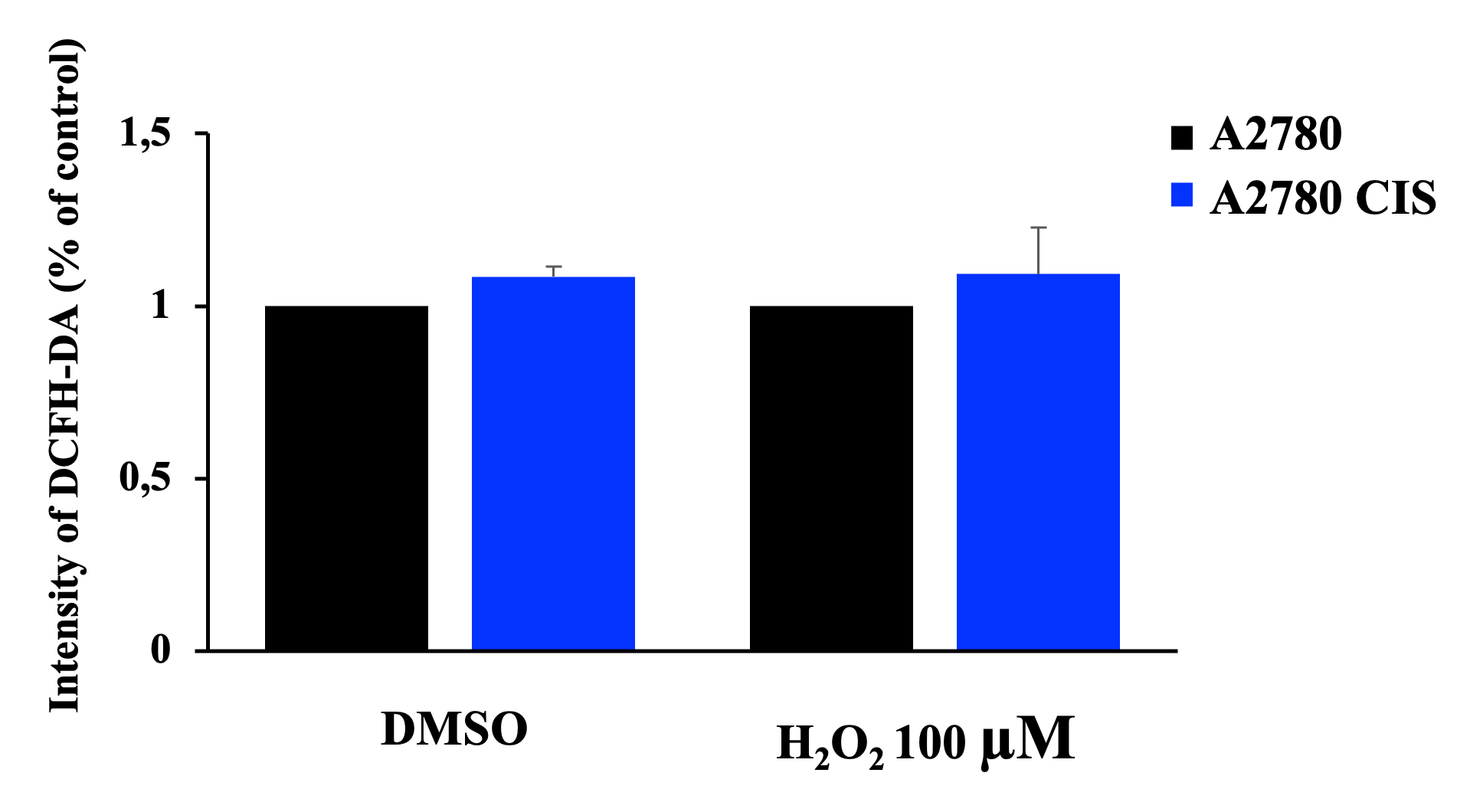


**Figure S1: Quantification of ROS production by 2’, 7’-dichlorodihydrofluorescein diacetate (DCFH-DA) staining.** A2780 and A2780 CIS cells were treated with 100 μM H_2_O_2_ for 4h and the intensity of fluorescence was measured microplate reader (Victor X5, Perkin Elmer). Data represent the mean ± SEM of at least three independent experiments.

**
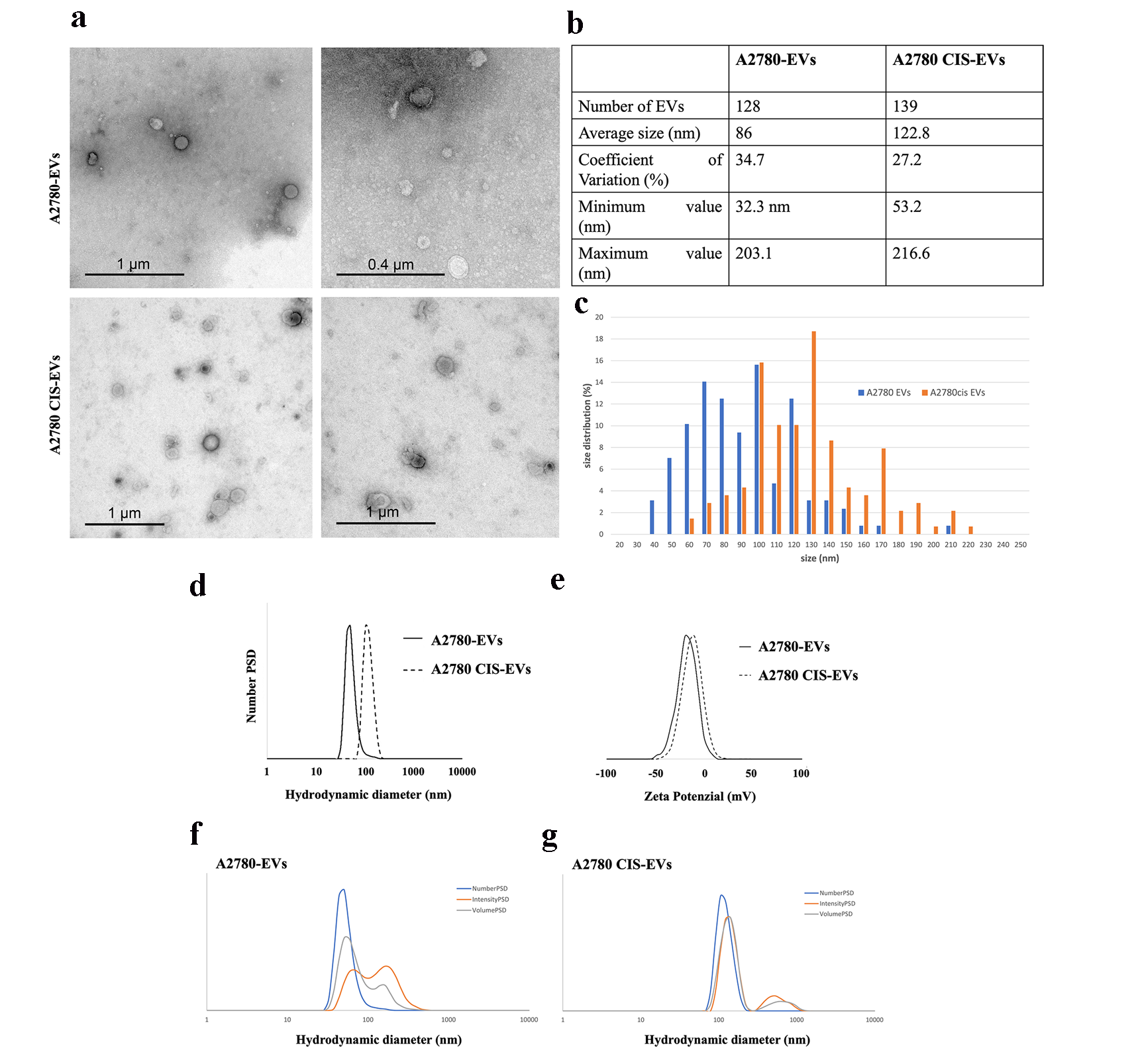
**

**Figure S2: TEM and DLS analyses of purified EVs.** (**a**) Large field pictures resulting from TEM analysis of samples of EVs coming from A2780 and A2780 CIS cells. (**b, c**) Table and relative histogram on the analysis of EV size obtained by TEM. (**d-g**) DLS, in order to evaluate the hydrodynamic size of the vesicles and their distribution. (**d**) Volume Particle Size Distribution (PSD) and (**e**) the ζ-potential analyses of the EVs were performed by DLS. In the two graphs below, three different distribution modalities (Number, Intensity and Volume Particle Size Distribution, PSD) are reported for A2780 EVs (**f**) and A2780 CIS EVs (**g**).

**
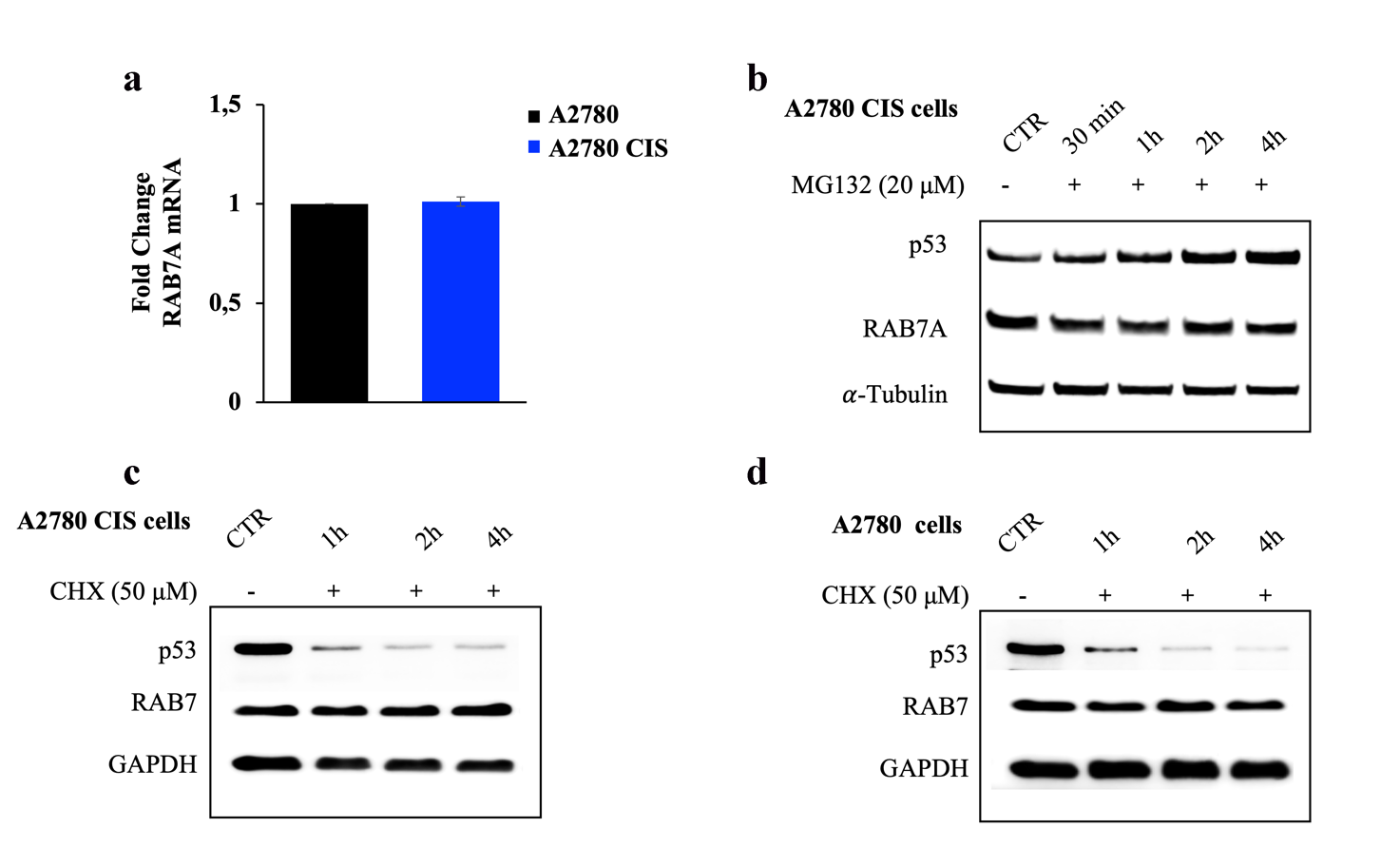
**

**Figure S3: Analysis of transcriptional and translational changes and alteration of RAB7 degradation.** (**a**) qRT-PCR was performed on A2780 and A2780 CIS cells and the amount of RAB7A mRNA was quantified relative to GAPDH. (**b**) A2780 CIS cells were subject to MG132 (20 µM) treatment for 30 min, 1, 2, and 4 h, and the RAB7 level was analyzed compared to non-treated cells (CTR). Α-tubulin was used as a housekeeping protein and p53 protein as a positive control of MG132. (**c-d**) A2780 CIS and A2780 cells were subject to cycloheximide (CHX, 50 µM) treatment to 1, 2, and 4h, and the RAB7 level was analyzed compared to non-treated cells (CTR). GAPDH was used as a housekeeping protein and p53 protein as a positive control of CHX.

**Table S1: Technical specifications of the primary antibodies for Western Blotting, primer sequences for quantitative Real time PCR.**

| **Primary antibodies** | **Manufacturer** | **Catalog Number** | **Species** | **Diluition** | **Detected Band MW (kDa)** |
| --- | --- | --- | --- | --- | --- |
| ATP5A (complex V) UQCRC2 (complex III)  MTCOI (complex IV)  SDHB (complex II) NDUFB8 (complex I) | Abcam (Cambridge, MA, USA) | ab110413 | Mouse | 1:500 | 55  48  40  30  20 |
| COMPLEX II Antibody Cocktail (SDHA/SDHB) | Abcam (Cambridge, MA, USA) | ab110410 | Mouse | 1:500 | 70  30 |
| CD9 | Santa Cruz Biotechnology (Santa Cruz, CA, USA) | sc-13118 | Mouse | 1:200 | 24 |
| CD63 | Santa Cruz Biotechnology (Santa Cruz, CA, USA) | sc-5275 | Mouse | 1:200 | 60  30 |
| CD81 | Santa Cruz Biotechnology (Santa Cruz, CA, USA) | sc-166029 | Mouse | 1:200 | 22 |
| NDUFS3 | Santa Cruz Biotechnology (Santa Cruz, CA, USA) | sc-374282 | Mouse | 1:1000 | 25 |
| Flotillin-1 | Santa Cruz Biotechnology (Santa Cruz, CA, USA) | sc-74566 | Mouse | 1:200 | 48 |
| Alix | Cell Signaling Technology | #2171 | Mouse | 1:1000 | 95 |
| RP-S6 | Santa Cruz Biotechnology (Santa Cruz, CA, USA) | sc-74459 | Mouse | 1:1000 | 32 |
| TSG-101 | Abcam (Cambridge, MA, USA) | ab30871 | Rabbit | 1:1000 | 50 |
| RAB7A | Santa Cruz Biotechnology (Santa Cruz, CA, USA) | sc-376362 | Mouse | 1:500 | 25 |
| RAB4A | Santa Cruz Biotechnology (Santa Cruz, CA, USA) | sc-312 | Rabbit | 1:200 | 25 |
| RAB5A | Santa Cruz Biotechnology (Santa Cruz, CA, USA) | sc-309 | Rabbit | 1:200 | 25 |
| RAB9A | Abcam (Cambridge, MA, USA) | ab2810 | Mouse | 1:1000 | 22 |
| RAB27A | Abcam (Cambridge, MA, USA) | ab55667 | Mouse | 1:1000 | 25 |
| LAMP-1 | Abcam (Cambridge, MA, USA) | ab24170 | Rabbit | 1:4000 WB  1: 250 IF | 120 |
| β-Actin | Santa Cruz Biotechnology (Santa Cruz, CA, USA) | sc-47778 | Mouse | 1:5000 | 42 |
| RILP | Abcam (Cambridge, MA, USA) | ab128616 | Rabbit | 1:1000 | 44 |
| ATP6-V1G1 | Abcam (Cambridge, MA, USA) | ab15853 | Chicken | 1:1000 | 13 |
| TFAM | Santa Cruz Biotechnology (Santa Cruz, CA, USA) | sc-376672 | Mouse | 1:1000 | 25 |
| PGC1 alpha | Abcam (Cambridge, MA, USA) | ab191838 | Rabbit | 1:1000 | 95 |
| PGC1beta | Abcam (Cambridge, MA, USA) | ab176328 | Rabbit | 1:1000 | 113 |
| NRF-1 | Santa Cruz Biotechnology (Santa Cruz, CA, USA) | sc-28379 | Mouse | 1:500 | 30 |
| Phospho-mTOR (Ser2448) | Cell Signaling Technology | #5536 | Rabbit | 1:1000 | 289 |
| mTOR | Cell Signaling Technology | #2983 | Rabbit | 1:1000  1:200 IF | 289 |
| Raptor | Cell Signaling Technology | #2280 | Rabbit | 1:1000 | 150 |
| Rictor | Cell Signaling Technology | #2114 | Rabbit | 1:1000 | 200 |
| GβL | Cell Signaling Technology | #3274 | Rabbit | 1:1000 | 37 |
| p-S6K (Thr421/Ser424) | Cell Signaling Technology | #9204 | Rabbit | 1:1000 | 75 |
| S6K | Cell Signaling Technology | #2708 | Rabbit | 1:1000 | 70 |
| p53 | Cell Signaling Technology | #2524 | Mouse | 1:1000 | 53 |
| Pink1 | Santa Cruz Biotechnology (Santa Cruz, CA, USA) | sc-517353 | Mouse | 1:500 | 66 |
| Parkin | Santa Cruz Biotechnology (Santa Cruz, CA, USA) | sc-30130 | Rabbit | 1:500 | 50 |
| LC3B-I  LC3B-II | Nanotools | 0231-100 | Mouse | 1:500 | 18  16 |
| TOMM20 | Abcam (Cambridge, MA, USA) | ab186734 | Rabbit | 1:1000 WB  1:250 IF | 16 |
| SOD1 | Santa Cruz Biotechnology (Santa Cruz, CA, USA) | sc-17767 | Mouse | 1:1000 | 23 |
| SOD2 | Santa Cruz Biotechnology (Santa Cruz, CA, USA) | sc-137254 | Mouse | 1:1000 | 25 |
| Catalase | Santa Cruz Biotechnology (Santa Cruz, CA, USA) | sc-271242 | Mouse | 1:500 | 64 |
| PRX | Santa Cruz Biotechnology (Santa Cruz, CA, USA) | sc-137222 | Mouse | 1:500 | 25 |
| TFEB | Bethyl Laboratories | A303-673A | Rabbit | 1:1000 | 65 |
| HA-Tag | Santa Cruz Biotechnology (Santa Cruz, CA, USA) | sc-7392 | Mouse | 1:500 | 2 |
| GFP-Tag | Santa Cruz Biotechnology (Santa Cruz, CA, USA) | sc-9996 | Mouse | 1:1000 | 27 |
| GAPDH | Santa Cruz Biotechnology (Santa Cruz, CA, USA) | sc-25778 | Rabbit | 1:1000 | 37 |
| Histone H3 | Abcam (Cambridge, MA, USA) | ab1791 | Rabbit | 1:1000 | 17 |
| Hsp90 α/β | Santa Cruz Biotechnology (Santa Cruz, CA, USA) | sc-13119 | Mouse | 1:5000 | 90 |
| α-Tubulin | Santa Cruz Biotechnology (Santa Cruz, CA, USA) | sc-23948 | Mouse | 1:5000 | 55 |
| Vinculin | Sigma-Aldrich | V9131 | Mouse | 1:1000 | 120 |
| **Secondary antibodies** | **Manufacturer** | **Catalog Number** | **Conjugate** | **Applcation** | |
| Anti-mouse | Bio-Rad (Hercules, CA, USA) | #1706516 | HRP | WB | |
| Anti-rabbit | Bio-Rad (Hercules, CA, USA) | #1706515 | HRP | WB | |
| Anti-chicken | Abcam (Cambridge, MA, USA) | ab6877 | HRP | WB | |
| Anti-rabbit | Life Technologies (Carlsbad, CA, USA). | A21206 | Alexa Fluor 488 | IF | |
| Anti-rabbit | Life Technologies (Carlsbad, CA, USA). | A11011 | Alexa Fluor 568 | IF | |
| **PRIMERS** | | | | | |
| **Gene** | **Primer sequence 5′-3′** | | | | |
| RAB7 | Forward 5′-CACAATAGGAGCTGACTTTCTGACC-3′  Reverse 5′-GTTCCTGTCCTGCTGTGTCCCATATC-3′ | | | | |
| Rplp0 | Forward 5′- TCGACAATGGCAGCATCTAC-3′  Reverse 5′- ATCCGTCTCCACAGACAAGG-3′ | | | | |

Abbreviations: ATP5A, adenosine triphosphate Synthase Subunit 5 Alpha 5A; MTCOI, mitochondrial cytochrome C oxidase subunit I; MTCOII, mitochondrial cytochrome C oxidase subunit II; NDUFB8, NADH:ubiquinone oxidoreductase subunit B8; SDHA, succinate dehydrogenase complex flavoprotein subunit A; SDHB, succinate dehydrogenase complex iron sulphur subunit B; UQCRC2, ubiquinol-cytochrome C reductase core protein 2; NDUFS3, NADH:Ubiquinone Oxidoreductase Core Subunit S3; RPS6, Ribosomal Protein S6; TSG 101, Tumor Susceptibility Gene 101; RAB7A, Ras-related brain-7A; LAMP-1, Lysosomal Associated Membrane Protein 1; RILP, Rab-interacting lysosomal protein; ATP6-V1G1, ATPase H+ Transporting V1 Subunit G1; TFAM, mitochondrial transcription factor A; PGC-1α and PGC-1β, proliferator-activated receptor gamma coactivator-1 alpha and beta; NRF1, Nuclear Respiratory Factor 1; mTOR, mammalian target of rapamycin; GβL, G protein beta subunit-like; p-S6K, Phospho-p70 S6 Kinase; S6K, p70 S6 Kinase; LC3, Microtubule-associated protein 1A/1B-light chain 3; SOD1, Superoxide Dismutase 1; SOD2, Superoxide Dismutase 2; PRX, peroxiredoxin; TFEB, Transcription Factor EB; GAPDH, glyceraldehyde-3-phosphate dehydrogenase; Hsp90 α/β, heat shock protein 90.
